# TFEB coordinates autophagosome biogenesis and ribophagy during starvation via SQSTM1

**DOI:** 10.1126/sciadv.aea9302

**Published:** 2026-01-01

**Authors:** Maria Iavazzo, Laura Cinque, Sophie Levantovsky, Castrese Morrone, Jlenia Monfregola, Andrea Raimondi, Elena Polishchuk, Rossella De Cegli, Diego Carrella, Edoardo Nusco, Luigi Ferrante, Francesca Sacco, Lisa B. Frankel, Christian Behrends, Carmine Settembre

**Affiliations:** ^1^ Telethon Institute of Genetics and Medicine (TIGEM), Pozzuoli, Italy.; ^2^ Department of Clinical Medicine and Surgery, Federico II University, Naples, Italy.; ^3^ Munich Cluster for Systems Neurology, Medical Faculty, Ludwig-Maximilians-Universität München, Munich, Germany.; ^4^ Institute for Research in Biomedicine (IRB), Bellinzona, Switzerland.; ^5^ Department of Biology, University of Rome Tor Vergata, Via delle Ricerca Scientifica 1, 00133 Rome, Italy.; ^6^ Danish Cancer Institute, 2100 Copenhagen, Denmark.; ^7^ Biotech Research and Innovation Centre, University of Copenhagen, 2200 Copenhagen, Denmark.

## Abstract

(Macro)autophagy is a conserved cellular degradation pathway that delivers substrates to lysosomes via autophagosomes. Among various physiological stimuli, nutrient starvation is the most potent inducer of autophagy. In response to starvation, transcription factor EB (TFEB) is activated and up-regulates a broad set of autophagy-related genes. However, the mechanisms by which TFEB promotes autophagosome biogenesis remain incompletely understood. Here, we demonstrate that TFEB-mediated transcriptional induction of *sequestosome 1* (SQSTM1; p62) triggers the formation of SQSTM1-positive bodies that recruit essential autophagy factors, thereby initiating autophagosome biogenesis. Genetic disruption of TFEB-dependent SQSTM1 regulation markedly impairs starvation-induced autophagy, underscoring the critical role of the TFEB-SQSTM1 axis in the autophagic response to nutrient stress. Furthermore, we show that these SQSTM1 bodies contain ubiquitinated ribosomal proteins and that TFEB promotes ribosomal protein ubiquitination by inducing the E3 ubiquitin ligase ZNF598. Collectively, our findings uncover a transcriptionally coordinated mechanism that regulates both autophagosome biogenesis and substrate ubiquitination, facilitating efficient cargo clearance during starvation-induced autophagy.

## Teaser

During starvation, TFEB activates SQSTM1-dependent autophagy to degrade ubiquitinated ribosomal components.

## INTRODUCTION

Nutrient starvation is the most potent known physiological inducer of autophagy. The inhibition of the mammalian target of rapamycin complex 1 (mTORC1) kinase by nutrient depletion activates the ULK1 (Unc-51–like kinase 1) autophagy initiation complex, which phosphorylates several autophagy proteins to initiate autophagosome biogenesis (*1*). In addition, mTORC1 inhibition leads to dephosphorylation and nuclear translocation of transcription factor EB (TFEB) and TFE3, transcriptional regulators of lysosomal biogenesis and autophagy genes (*2*–*7*). Consequently, the inhibition of mTORC1 during starvation enhances autophagosome biogenesis at both transcriptional and posttranscriptional levels. Intriguingly, constitutive activation of TFEB and/or TFE3, as observed in pancreatic and renal cancers, can induce autophagy independently of mTORC1 activity (*8*–*10*). This observation suggests that transcriptional up-regulation of key autophagy gene(s) is, by itself, sufficient to trigger autophagy. Elucidating this phenomenon will advance our understanding of how transcriptional regulation controls autophagy and provide key insights into the pathogenic mechanisms underlying TFEB- and TFE3-driven malignancies.

A critical step in autophagy is substrate selection, which can occur through bulk, indiscriminate engulfment of cellular components or through tailored mechanisms of cargo recognition. Key regulators of cargo selection are autophagy cargo receptors (*11*). One of the best examples of selective autophagy is aggrephagy, the removal of protein aggregates via the autophagy pathway (*12*). Aggrephagy is initiated by the autophagy cargo receptor P62/sequestosome 1 (SQSTM1), which condensates ubiquitinated proteins within large bodies (also known as SQSTM1 puncta) (*13*). The association of neighbor of BRCA1 gene 1 (NBR1) with SQSTM1 bodies, along with Tax1 binding protein 1 (TAX1BP1), promotes the subsequent recruitment of key autophagy-initiating factors, such as the FAK family kinase–interacting protein of 200 kDa (FIP200) subunit of the ULK1 complex and the TANK binding kinase 1 (TBK1) kinase, thereby promoting autophagosome biogenesis around the SQSTM1 condensate (*14*, *12*).

Ubiquitination is a posttranslational modification involving the covalent attachment of ubiquitin molecules to specific target proteins. This process occurs in three sequential enzymatic steps: activation of ubiquitin by an E1 enzyme, its transfer to an E2 conjugating enzyme, and lastly, ligation to the substrate protein by an E3 ligase (*15*). Ubiquitination commonly functions as a signal for proteasomal degradation and facilitates substrate recognition by selective autophagy receptors, such as SQSTM1 (*16*).

Ubiquitination of ribosomal subunit proteins is a hallmark of ribosome-associated quality control (RQC), a pathway activated upon ribosomal stalling and collision resulting from translational errors (*17*). Ubiquitinated ribosomes may subsequently be deubiquitylated and recycled or targeted for lysosomal degradation (*18*). Notably, a fraction (5 to 10%) of ribosomes is specifically degraded by autophagy (ribophagy) during nutrient starvation (*19*), a process mediated, at least in part, by dedicated ribophagy receptors such as nuclear FMR1-interacting protein 1 (NUFIP1) and ribosomal protein L12 (RPL12), which directly interact with LC3 proteins to facilitate ribosomal incorporation into autophagic vesicles (*20*, *21*). Zinc finger protein 598, E3 ubiquitin ligase (ZNF598) functions as a bona fide E3 ubiquitin ligase and initiator of the ribosome-associated quality control pathway, recognizing collided ribosomes and catalyzing the ubiquitination of the small ribosomal subunit proteins ribosomal protein S10 (RPS10), RPS20, RPS3, and RPS2 (*18*, *22*–*24*). Additional E3 ligases subsequently modify other ribosomal subunits. In contrast, the deubiquitinating enzyme ubiquitin-specific peptidase 10 (USP10) promotes the deubiquitylation and recycling of RPS proteins (*18*). Loss of USP10, as observed during oncogene-induced senescence, enhances ribosomal ubiquitination and drives selective ribosome degradation through SQSTM1-mediated autophagy (*25*). However, a potential role for ZNF598 in starvation-induced ribophagy remains unexplored.

In this study, we investigated the molecular mechanism by which TFEB activation induces autophagy. We demonstrate that TFEB activation promotes the formation of SQSTM1-positive bodies, which enhance autophagy induction during nutrient starvation. We further identify the E3 ubiquitin ligase ZNF598 as a TFEB-responsive gene that is up-regulated during starvation and facilitates the ubiquitination of ribosomal proteins and their sequestration into SQSTM1 bodies. These findings reveal a previously unrecognized transcriptionally coordinated mechanism whereby the co-regulation of an autophagy receptor (SQSTM1) and its corresponding substrate ubiquitination (mediated by ZNF598) synchronizes autophagosome biogenesis and substrate sequestration during starvation-induced autophagy.

## RESULTS

### TFEB overexpression activates aggrephagy via SQSTM1 induction

**Fig. 1.**
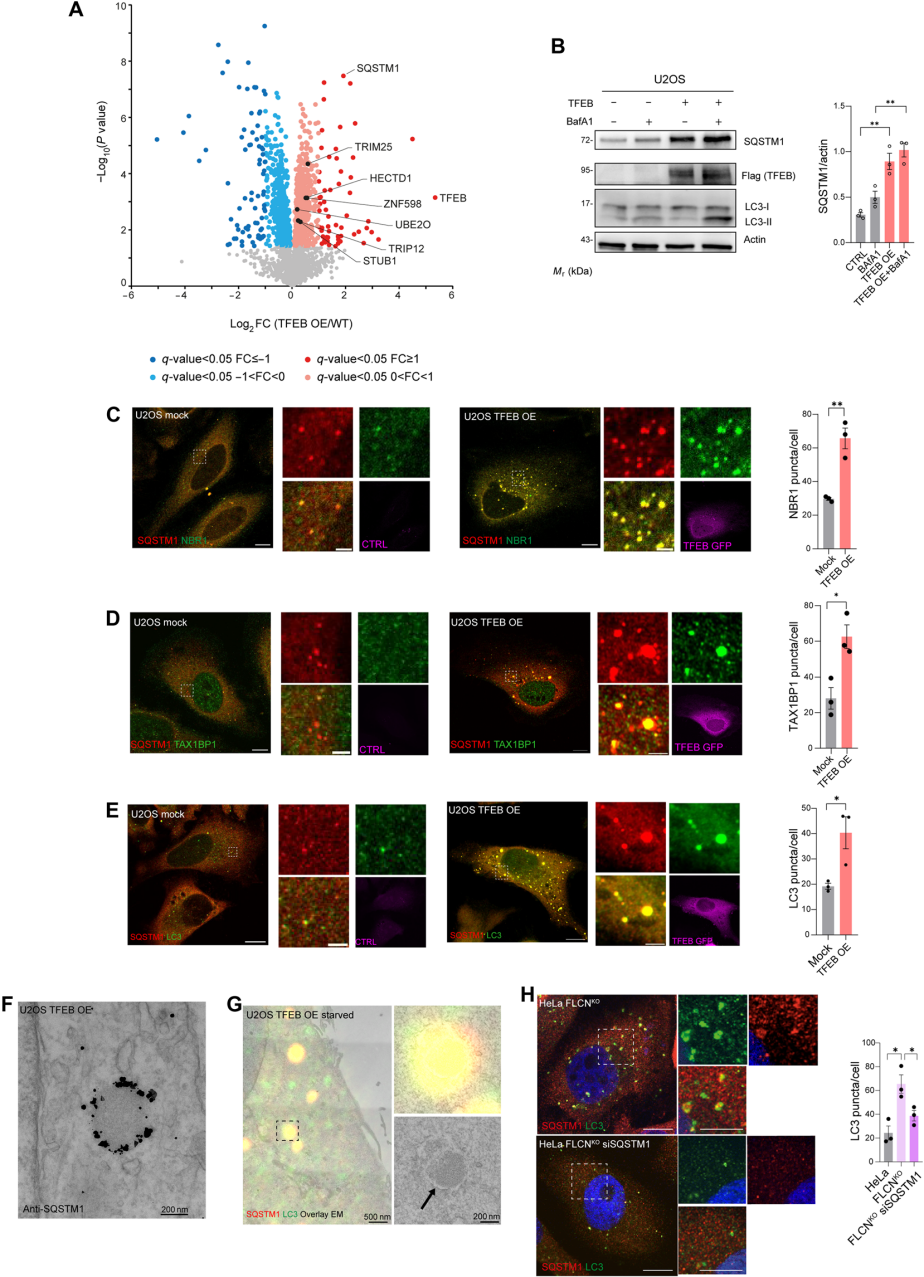
TFEB induces autophagy via SQSTM1 puncta formation. (**A**) Volcano plots of proteins from whole-cell proteomics in TFEB-3xFlag versus wild-type (WT) U2OS cells. Significantly altered proteins: dark [FDR < 0.05, log_2_ fold change (FC) > |1|] and light (FDR < 0.05, 0 < |log_2_FC| < |1|) red/blue (FDR-corrected two-sided *t* test, *N* = 4). E3 ligases are highlighted in black (table S1). (**B**) Western blot of indicated proteins in control and TFEB-3xFlag U2OS cells (*N* = 3) with or without BafA1 (200 nM, 4 hours). Quantification normalized to β-actin; means ± SEM. One-way ANOVA: *P* = 0.0003. Sidak’s test: **P < 0.005. *M*_r_, relative molecular mass. (**C** to **E**) Coimmunofluorescence of SQSTM1 (red) with (C) NBR1, (D) TAX1BP1, or (E) LC3B (green) in CTRL and TFEB-GFP (purple) cells. Scale bars, 10 μm (insets, 2 μm). Quantification of puncta per cell; means ± SEM (*N* = 3, *n* = 24 to 30 cells). Student’s unpaired *t* test: **P < 0.005 for NBR1, *P < 0.05 for TAX1BP1 and LC3B. (**F**) Electron micrograph of TFEB-GFP U2OS cell labeled for SQSTM1 (nanogold). (**G**) CLEM of TFEB-GFP U2OS cells starved in HBSS (2 hours) and labeled for SQSTM1 (red) and LC3B (green). The arrow indicates membranes surrounding an SQSTM1- and LC3B-positive structure. (**H**) Coimmunofluorescence of SQSTM1 (red) and LC3B (green) in *FLCN*^*KO*^ HeLa cells ± *siSQSTM1* (100 nM for 48 hours). Scale bars, 10 μm (insets, 5 μm). Quantification of LC3B puncta per cell; means ± SEM (*N* = 3, *n* = 30 cells). Student’s unpaired *t* test: *P < 0.05.

Mass spectrometry (MS)–based whole-cell protein abundance profiling in U2OS cells stably overexpressing TFEB-3xFlag identified the autophagy receptor P62/SQSTM1 as the most significantly up-regulated autophagy-related protein induced by TFEB (Fig. 1A). SQSTM1 protein levels were further increased after blocking lysosomal degradation with the vacuolar-type adenosine triphosphatase (v-ATPase) inhibitor bafilomycin A1 (BafA1), indicating that SQSTM1 production is increased in TFEB-overexpressing cells (Fig. 1B). TFEB induced the transcription of *SQSTM1*, whereas the transcription of other tested autophagy receptors was not influenced, except for reticulophagy regulator 1 (RETREG1, also known as FAM134B) (fig. S1A), as we reported recently (*26*). TFEB overexpression promoted the formation of SQSTM1 puncta, whose number increased by BafA1 treatment (fig. S1B), suggesting that they are degraded by the lysosome-autophagy pathway.

SQSTM1 puncta induced by TFEB are positive for NBR1 (88 ± 1.83%) and TAX1BP1 (69 ± 7.44%) and are decorated with early and late autophagy markers, including WD repeat domain, phosphoinositide-interacting 2 (WIPI-2; 48 ± 6.8%) and double-FYVE domain–containing protein 1 (DFCP1; 52 ± 5.5%), and with microtubule-associated protein 1 light chain 3β (LC3B; 77 ± 2.12%) (Fig. 1, C to E, and fig. S1, C and D). Immuno–electron microscopy (IEM) demonstrated that SQSTM1 decorates round-shaped structures, which, by correlative light transmission EM (CLEM) analysis, were found positive for LC3B (Fig. 1, F and G). We also analyzed autophagy in cells lacking the *Folliculin* gene (*FLCN*^*KO*^), which exhibit constitutive nuclear localization of TFEB and TFE3 (*10*). Loss-of-function mutations in *FLCN* lead to Birt-Hogg-Dubé syndrome in humans, a rare inherited disease characterized by skin tumors and renal cysts (*10*). Despite having sustained mTORC1 signaling (*10*), *FLCN*^*KO*^ cells display increased *SQSTM1* expression and a significant induction of LC3B-positive vesicles compared to control cells. A total of 57 ± 4.3% of SQSTM1-positive puncta colocalize with LC3B in *FLCN*^*KO*^ cells (Fig. 1H and fig. S1E).

**Fig. 2.**
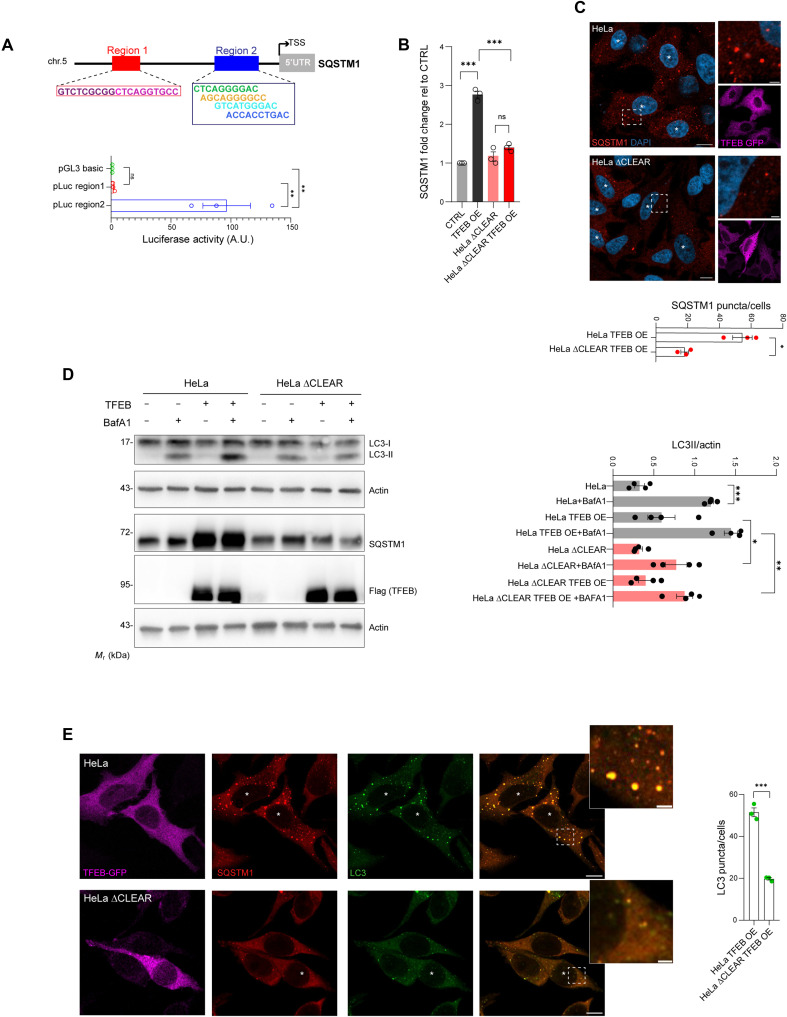
Deletion of TFEB binding sites in the *SQSTM1* promoter impairs TFEB-induced autophagy. (**A**) Schematic representation of the *SQSTM1* promoter with putative TFEB binding sites. Regions 1 and 2 were cloned into the pGL3-basic luciferase reporter plasmid, and the luciferase activity was determined. Means ± SEM of *N* = 3. One-way ANOVA: *P* = 0.0016. Sidak’s test: **P < 0.005. ns, not significant. TSS, transcription start site; 5′UTR, 5′ untranslated region; A.U., arbitrary units. (**B**) qRT-PCR analysis of *SQSTM1* expression in CTRL and ΔCLEAR HeLa cells with or without TFEB overexpression (TFEB OE). Fold change normalized to *HPRT* and expressed relative to CTRL. Means ± SEM of *N* = 3. One-way ANOVA, *P* < 0.0001. Sidak’s test: ***P < 0.0001. (**C**) Immunofluorescence of SQSTM1 (red) and TFEB-GFP (purple) in WT and ΔCLEAR HeLa cells overexpressing TFEB-GFP. Nuclei stained with DAPI (blue). Scale bars, 10 μm (insets, 2 μm). Quantification of SQSTM1 puncta per cell; means ± SEM (*N* = 3, *n* = 40 cells). Student’s unpaired *t* test: *P < 0.05. (**D**) Western blot analysis of indicated proteins in WT and ΔCLEAR HeLa cells infected with TFEB3xFlag, with or without BafA1 (200 nM, 4 hours). Quantification of LC3BII normalized to β-actin; means ± SEM (*N* = 4). One-way ANOVA: ***P < 0.0001. Sidak’s test: ***P < 0.0005; **P < 0.005; *P < 0.05. (**E**) Coimmunofluorescence staining of LC3B (green), SQSTM1 (red), and TFEB-GFP (purple) in WT and ΔCLEAR HeLa cells overexpressing TFEB-GFP. Scale bars, 10 μm. Quantification of LC3B puncta per cell; means ± SEM [*N* = 3, *n* = 41 (HeLa) and *n* = 40 (ΔCLEAR HeLa) cells]. Student’s unpaired *t* test: ***P = 0.001.

Next, we asked whether SQSTM1 induction was associated with an increased number of LC3B-positive vesicles in cells with enhanced TFEB activity. Silencing of *SQSTM1* in cells with TFEB overexpression blunted LC3BII induction, and similarly, *SQSTM1* silencing in *FLCN*^*KO*^ cells significantly reduced the number of LC3B-positive vesicles (Fig. 1H and fig. S1F). These data suggest that TFEB activation or overexpression promotes autophagosome biogenesis at least in part via SQSTM1 induction. To test this hypothesis, we generated a cell line in which *SQSTM1* expression was unresponsive to TFEB activation. Through computational analysis of available chromatin immunoprecipitation (IP) sequencing (ChIP-seq) data (*27*), we identified seven highly scoring putative TFEB binding sites (CLEAR sites) in the SQSTM1 promoter (fig. S2) clustered in two different promoter regions, which we named regions 1 and 2 (Fig. 2A). Luciferase assays demonstrated that region 2 exhibited the highest responsiveness to TFEB (Fig. 2A). Using CRISPR-Cas9 technology, we replaced the endogenous genomic region 2 with the one that lacks the CLEAR sites in HeLa cells (∆CLEAR HeLa). ∆CLEAR HeLa cells exhibited normal levels of *SQSTM1* at the steady state, but no SQSTM1 transcriptional, protein, or puncta induction was observed upon TFEB overexpression (Fig. 2, B to D). The ability of TFEB to induce autophagy was inhibited in ∆CLEAR HeLa cells, as demonstrated by the Western blot analysis of LC3BII levels and confocal microscopy analysis of the number of LC3B-positive puncta (Fig. 2, D and E). These data suggest that induction of SQSTM1 plays a crucial role in the regulation of TFEB-mediated autophagy.

### Transcriptional induction of *SQSTM1* promotes starvation-induced autophagy

**Fig. 3.**
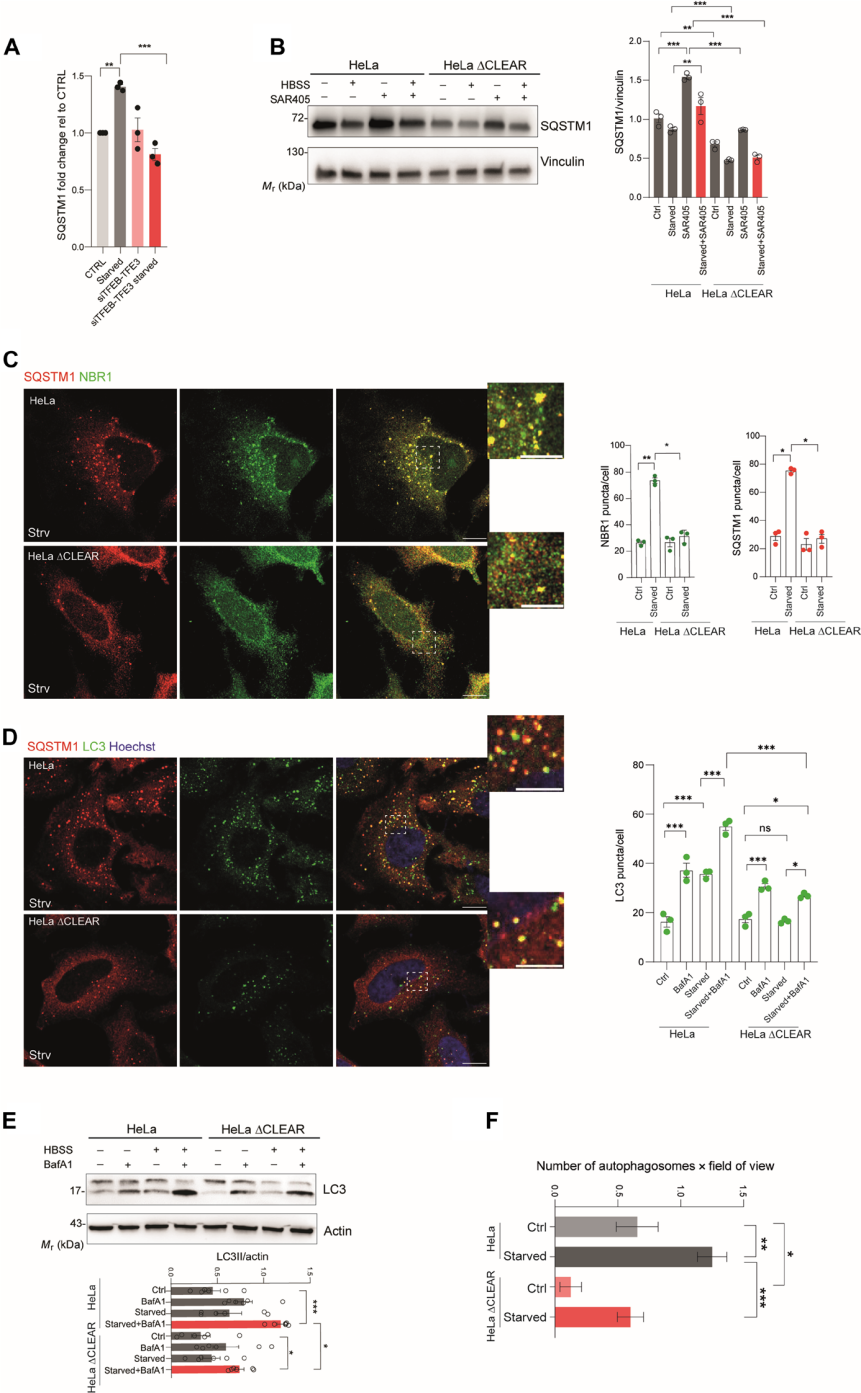
Deletion of TFEB binding sites in the SQSTM1 promoter impairs starvation-induced autophagy. (**A**) qRT-PCR of *SQSTM1* in CTRL and in *siTFEB-TFE3* HeLa cells ± HBSS (4 hours). Fold change normalized to *HPRT*; means ± SEM (*N* = 3). ANOVA, *P* = 0,0007. Sidak’s test: ***P = 0.0004, **P = 0.005. (**B**) Western blot of SQSTM1 in WT and ΔCLEAR HeLa cells ± HBSS (4 hours) and ± SAR405 (10 μM, 4 hours). Quantification normalized to vinculin; means ± SEM (*N* = 3). ANOVA: ***P < 0.0001. Sidak’s test: ***P < 0.0005; **P < 0.005. (**C**) Coimmunofluorescence of SQSTM1 (red) and NBR1 (green) in WT and ΔCLEAR HeLa cells ± HBSS (2 hours). Scale bars, 10 μm (insets, 5 μm). Quantification of puncta per cell; means ± SEM (*N* = 3, *n* = 30 cells). ANOVA: **P = 0.0012 and **P = 0.0034. Sidak’s test: **P < 0.005; *P < 0.05. (**D**) Coimmunofluorescence of SQSTM1 (red) and LC3B (green) in WT and ΔCLEAR HeLa cells ± HBSS (2 hours). Scale bars, 10 μm (insets, 5 μm). Quantification of LC3B puncta per cell; means ± SEM (*N* = 3, *n* = 30 cells). ANOVA: ***P < 0.0001. Sidak’s test: ***P < 0.0005; **P < 0.005. (**E**) Western blot of LC3B in WT and ΔCLEAR HeLa ± HBSS (4 hours) and ± BafA1 (200 nM, 4 hours). Quantification of LC3BII/β-actin; means ± SEM (*N* = 6). ANOVA: ***P < 0.0001. Sidak’s test: ***P < 0.0005; *P < 0.05. (**F**) Transmission EM of autophagosomes in WT and ΔCLEAR HeLa ± HBSS (2 hours). Quantification per field of view; means ± SEM. ANOVA: ***P < 0.0001. Sidak’s test: ***P < 0.0001; **P < 0.005; *P < 0.05 (*n* = 26, fed; *n* = 40, starved; *n* = 16, ΔCLEAR fed; *n* = 30, ΔCLEAR starved).

TFEB is rapidly activated upon nutrient starvation, becoming fully localized to the nucleus within 30 to 60 min. Cells lacking *TFEB* exhibit defects in starvation-induced autophagy. Thus, we asked whether TFEB contribution to autophagy induction during starvation was, at least in part, mediated by the induction of *SQSTM1*. Starvation induces the transcriptional induction of *SQSTM1*, and this effect is mediated by TFEB, given that the starvation response was blunted in cells in which *TFEB* and its paralog *TFE3* were silenced (*siTFEB-TFE3*) (Fig. 3A). Starvation induced SQSTM1 degradation in both control and ∆CLEAR HeLa cells. However, blocking autophagy with the vacuolar protein sorting 34 (VPS34) inhibitor SAR405 led to higher SQSTM1 levels in control cells compared to ∆CLEAR HeLa cells during starvation (Fig. 3B). This suggests that SQSTM1 is produced de novo during starvation, consistent with previous results (*28*), and that this is mediated by TFEB/TFE3 transcriptional activity. During starvation, the number of SQSTM1 puncta that are also positive for NBR1 increased in control but not in ∆CLEAR HeLa cells (Fig. 3C), suggesting that their formation is, at least in part, due to the transcriptional induction of *SQSTM1*. Starvation with Hanks’ balanced salt solution (HBSS) induced autophagosome biogenesis in HeLa cells, and we found that 42.5 ± 7.1% of the LC3B vesicles were also positive for SQSTM1. Notably, starvation-induced autophagy was defective in ∆CLEAR HeLa cells compared to the control cell line, as demonstrated by the reduced number of LC3B-positive vesicles and of LC3B lipidation in the presence of BafA1 (Fig. 3, D and E). Furthermore, EM analysis in control and ∆CLEAR HeLa cells cultured in nutrient-rich and starvation media confirmed a reduction in the number of bona fide autophagosomes, defined as double-membrane vesicles containing undigested cytosolic content, in starved ∆CLEAR compared to control HeLa cells (Fig. 3F and fig. S3). Overexpression of SQSTM1 in ∆CLEAR HeLa as well as in *TFEB/TFE3*^*dKO*^ cells markedly rescued the autophagy defects, as demonstrated by the quantification of LC3B-positive vesicles (fig. S4, A and B). Conversely, *SQSTM1* silencing reduced starvation-induced accumulation of LC3BII in HeLa cells in the presence of BafA1 (fig. S4C). Collectively, these data suggest that the transcriptional induction of *SQSTM1* sustains autophagy during starvation.

**Fig. 4.**
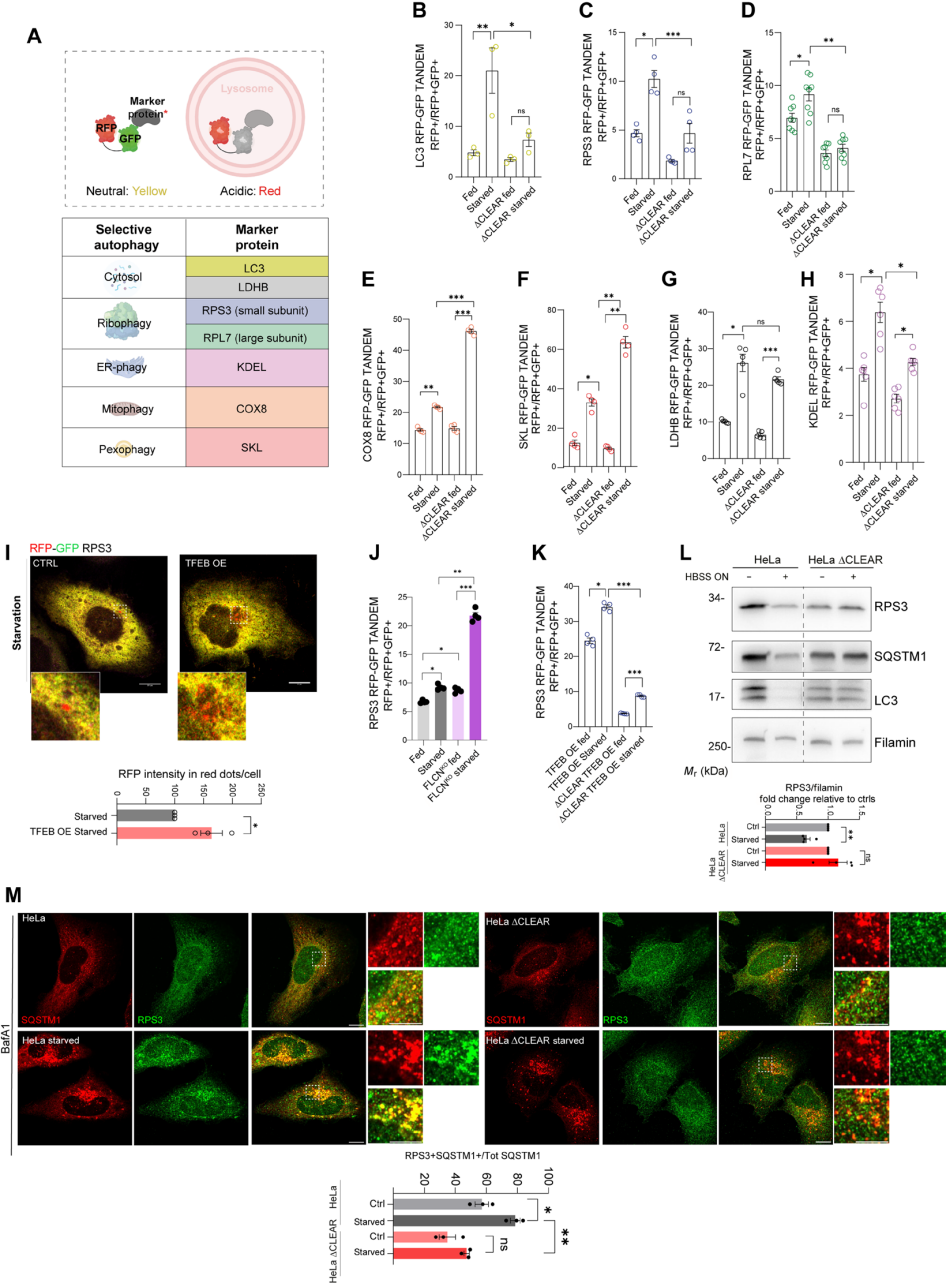
TFEB promotes ribosome degradation via SQSTM1. (**A**) Schematic of the tandem selective autophagy reporter in which eGFP (enhanced GFP) fluorescence, but not RFP, is quenched at acidic pH. Reporters used are listed in the table. (**B** to **H**) FACS analysis of tandem reporter assays in WT and ΔCLEAR HeLa cells ± HBSS (16 hours). Reporters analyzed: LC3B (B), RPS3 (C), RPL7 (D), COX8 (E), SKL (F), LDHB (G), and KDEL (H). Red fluorescence shift indicates autophagic flux. Means ± SEM (*N* = 3 to 8). One-way ANOVA with Sidak’s test: *P < 0.05; **P < 0.005; ***P < 0.0005. (**I**) Fluorescence microscopy of the RPS3 reporter in CTRL and TFEB-3xFlag–overexpressing U2OS cells starved in HBSS (16 hours). Scale bars, 10 μm (insets, 2 μm). Quantification of RFP puncta intensity per cell relative to control; means ± SEM (*N* = 3, *n* = 29 cells per condition). Student’s unpaired *t* test: *P = 0.026. (**J** to **K**) FACS analysis of the RPS3 tandem reporter in WT and *FLCN*^KO^ or ΔCLEAR HeLa cells ± HBSS (16 hours) or overexpressing TFEB-3xFlag. Quantification as in (B) to (H); means ± SEM (*N* = 4). One-way ANOVA: *P* < 0.0001; Sidak’s test: *P < 0.05; **P < 0.005; ***P < 0.0005. (**L**) Western blot of indicated proteins in WT and ΔCLEAR HeLa ± HBSS. Filamin was used as a loading control. Means ± SEM of *N* = 3. Student’s unpaired *t* test: **P < 0.005. (**M**) Coimmunofluorescence of SQSTM1 (red) and RPS3 (green) in WT and ΔCLEAR HeLa cells ± HBSS (2 hours). Scale bars, 10 μm (insets, 5 μm). Quantification of RPS3^+^ and SQSTM1^+^ puncta as a percentage of total SQSTM1 puncta per cell; means ± SEM (*N* = 3, *n* = 29 cells per condition). One-way ANOVA: ***P = 0.0003; Sidak’s test: *P < 0.05; **P < 0.0005.

Next, we investigated the contribution of SQSTM1 induction to starvation-induced autophagy. To study substrate degradation during autophagy, we generated ∆CLEAR HeLa and parental cell lines stably expressing fluorescent probes [green fluorescent protein (GFP)–red fluorescent protein (RFP)] to monitor the lysosomal degradation of LC3B, ribosomes, mitochondria, peroxisomes, the endoplasmic reticulum, and the cytosol (Fig. 4A) (*29*). Consistent with the findings described above, we found that starvation-induced delivery of LC3B to the lysosome was reduced in ∆CLEAR HeLa compared to parental cell lines (Fig. 4B). We also observed that the degradation of the small (RPS3) and large (RPL7) ribosomal protein subunits was markedly inhibited in ∆CLEAR HeLa cells (Fig. 4, C and D). Conversely, starvation-induced mitophagy [cytochrome c oxidase subunit 8 (COX8)], pexophagy [Ser-Lys-Leu (SKL)], cytosolic autophagy [lactate dehydrogenase B (LDHB)], and endoplasmic reticulum (ER)–phagy [Lys-Asp-Glu-Leu (KDEL)] were not altered in ∆CLEAR HeLa cells compared to parental control (Fig. 4, E and H).

Ribophagy was induced in both TFEB-overexpressing cells and *FLCN*^KO^ HeLa cells compared to their corresponding controls (Fig. 4, I and J, and fig. S4D). However, TFEB overexpression failed to induce ribophagy in ∆CLEAR compared to control HeLa cells (Fig. 4K). Most notably, the starvation-induced degradation of endogenous RPS3 as well as of SQSTM1 was evident in control cells upon HBSS treatment, but this effect was impaired in ∆CLEAR HeLa cells (Fig. 4L). Airyscan super-resolution microscopy analysis demonstrated that RPS3 colocalized with the SQSTM1 puncta formed during TFEB overexpression (fig. S5A), as well as during starvation, in control but not in ∆CLEAR HeLa cell lines (Fig. 4M). Collectively, these observations suggest that the TFEB-mediated transcriptional induction of SQSTM1 promotes ribosome protein degradation via aggrephagy.

### TFEB induces ZNF598, promoting ribosome protein ubiquitination and ribophagy

SQSTM1 forms condensates with ubiquitinated cargo through its ubiquitin-associated domain (UBA)–mediated interaction with ubiquitin chains (*30*, *16*). SQSTM1-positive structures induced upon TFEB overexpression colocalize with ubiquitin in U2OS and HeLa cells, indicating that SQSTM1 bodies contain ubiquitinated cargo (fig. S5, B and C).

**Fig. 5.**
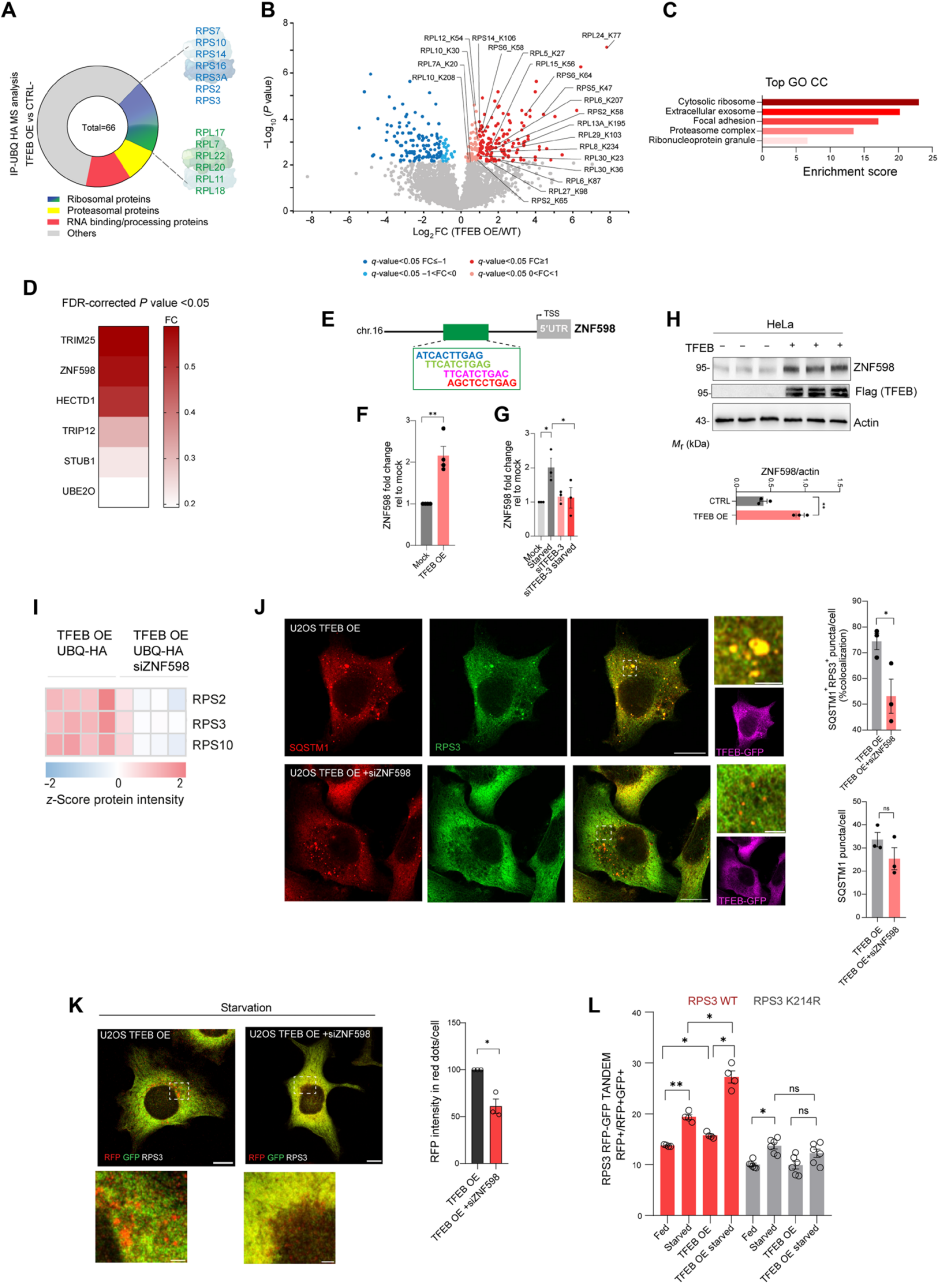
TFEB regulates ribosome ubiquitination via the E3 ubiquitin ligase ZNF598. (**A**) The UBQ-HA interactome in TFEB-3xFlag U2OS cells identified ribosomal proteins as a prominent category (table S2). (**B**) Volcano plot of ubiquitinated peptides by diGly proteomics in TFEB-3xFlag versus WT U2OS; significant changes in red/blue (FDR < 0.05, log_2_FC > 1 or < 1); two-sided *t* test, *N* = 4 (table S1). (**C**) Top five significant GO CC terms among 465 up-regulated proteins; enrichment score (ES) shown (FDR = 10% and ES > 1.5; table S5). (**D**) Heatmap of TFEB-regulated E3 ligase (FDR-corrected *t* test, *N* = 4; table S1). (**E**) *ZNF598* promoter schematic showing putative TFEB binding sites. (**F** and **G**) qRT-PCR of *ZNF598* in mock, TFEB-GFP, or *siTFEB-TFE3* U2OS ± HBSS (4 hours); normalized fold change (means ± SEM, *N* = 3 or 4; **P < 0.005; ANOVA: *P = 0.031; Sidak’s test: *P < 0.05). (**H**) Western blot of ZNF598 under indicated conditions; quantified versus β-actin (means ± SEM, *N* = 3), **P < 0.005. (**I**) Heatmap of significantly HA-ubiquitinated ribosomal proteins (S0 = 0.1, FDR < 0.05, *N* = 4) under indicated conditions (red: up-regulated; blue: down-regulated; table S3). (**J**) Coimmunofluorescence of SQSTM1 (red) and RPS3 (green) in TFEB-GFP ± *siZNF598*; scale bars, 10 μm (insets, 2 μm). Quantification: SQSTM1-RPS3 colocalization (%) and SQSTM1 puncta per cell (means ± SEM of *N* = 3, *n* = 38), *P < 0.05. (**K**) Fluorescence microscopy of the RPS3 reporter in starved (ON) TFEB-3xFlag ± *siZNF598*. Scale bars, 10 μm (insets, 2 μm). RFP intensity relative to scramble (means ± SEM of *N* = 3, *n* = 30), *P = 0.007. (**L**) FACS of the RPS3 WT or K214R reporter. Red fluorescence shift, means ± SEM [*N* = 4 (WT), *N* = 6 (K214R)]. ANOVA: *P* = 0.0006 (WT), *P* = 0.01 (K214R). Sidak’s test: *P < 0.05; **P < 0.005.

We explored the possibility that ribosomes are ubiquitinated and hence recognized by SQSTM1 structures. Ubiquitin-hemagglutinin (UBQ-HA) IP followed by MS identified 66 candidates that were enriched from samples isolated from TFEB-overexpressing cells compared to control cells (Fig. 5A). Gene ontology (GO) analysis clearly showed that ribosomal subunits were the most enriched category of ubiquitinated proteins (fig. S5D). Furthermore, proteomic analysis of diGly peptides revealed that TFEB overexpression strongly increased the ubiquitination of several ribosome subunits (Fig. 5, B and C). These results suggest the testable hypothesis that TFEB activation promotes ribosome ubiquitination.

Through quantitative whole-cell proteome analysis, we identified at least five E3 ubiquitin ligases significantly up-regulated in TFEB-overexpressing cells versus controls. We focused our attention on ZNF598 (Figs. 1A and 5D), a RING domain E3 ligase known for site-specific ubiquitination of ribosomal subunits. ZNF598 ubiquitinates ribosomal proteins in response to ribosome stalling (*24*, *31*), initiating ribosome quality control pathways that can culminate in the dissociation of the ribosomal subunits and their degradation by lysosomes (*18*). Prompted by these findings, we investigated the involvement of ZNF598 in TFEB- and starvation-mediated ribophagy. *ZNF598* has at least four putative TFEB binding sites (CLEAR) in the promoter region, and analysis of published ChIP-seq demonstrated TFEB binding to the promoter of *ZNF598* (Fig. 5E and fig. S6A). TFEB overexpression increased levels of ZNF598 mRNA and protein (Fig. 5, F and H, and fig. S6B). Starvation transcriptionally induced *ZNF598* levels in a TFEB-dependent fashion, given that this response was blunted in cells in which *TFEB* and its paralog *TFE3* were silenced (Fig. 5G and fig. S6C). These data suggest that *ZNF598* is transcriptionally regulated by TFEB and it is induced by starvation.

ZNF598 directly ubiquitinates RPS3, RPS10, RPS2, and RPS20 proteins (*18*). *ZNF598* silencing reduced the ubiquitination levels of RPS3, RPS2, and RPS10 in cells overexpressing TFEB (Fig. 5I and fig. S6D). It also hindered TFEB’s ability to promote the formation of RPS3 puncta that colocalize with SQSTM1 (Fig. 5J). Unfortunately, we could not evaluate the effects on RPS10, RPS2, and RPS20 on colocalization because we did not detect reliable immunofluorescence staining for these proteins. *ZNF598* silencing severely impaired RPS3 delivery to the lysosome in response to starvation (Fig. 5K). Furthermore, introducing a Lys^214^→Arg (K214R) substitution at the lysine residue ubiquitinated by ZNF598 (*18*) strongly inhibited the starvation- and TFEB-induced ribophagy of RPS3 (Fig. 5L and fig. S6E). Collectively, these data support the model by which ubiquitination of ribosome subunits by ZNF598 promotes their degradation via SQSTM1-dependent autophagy.

### Starvation-induced ribophagy in the liver is regulated by TFEB and SQSTM1

**Fig. 6.**
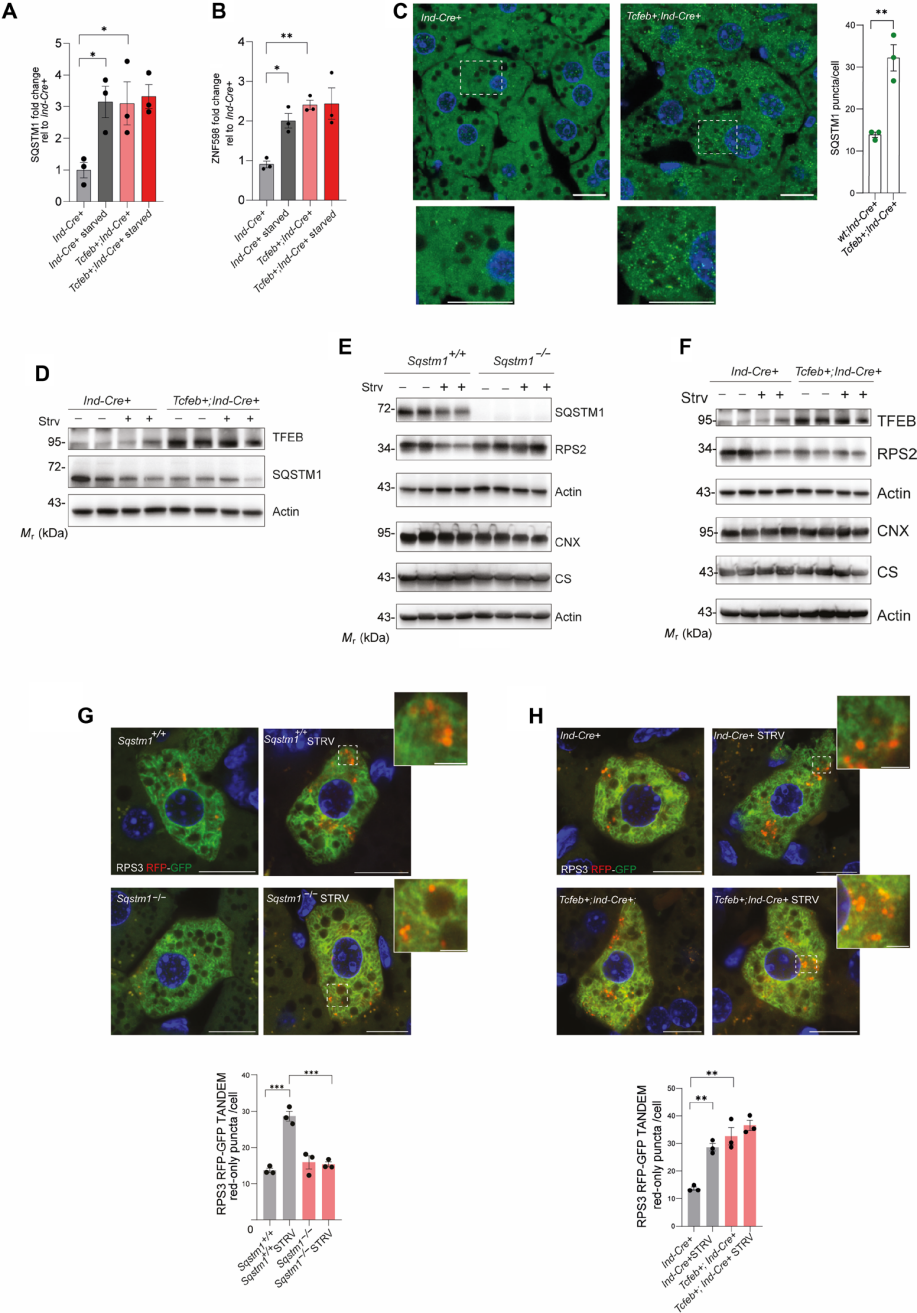
TFEB and SQSTM1 regulate ribophagy flux in the mouse liver. (**A**) qRT-PCR of *SQSTM1* in the liver from fed or 24-hour starved mice with indicated genotypes. Fold change relative to CTRL mice *(Ind-Cre*+), normalized to *cyclophilin* (means ± SEM of *N* = 3 mice per condition). One-way ANOVA: *P = 0.02. Sidak’s test: *P < 0.05. (**B**) qRT-PCR of *ZNF598* in the liver from fed or 24-hour starved mice with indicated genotypes. Fold change relative to CTRL mice (*Ind-Cre*+), normalized to *cyclophilin* (means ± SEM of *N* = 3 mice per condition). One-way ANOVA: **P < 0.005. Sidak’s test: **P < 0.005, *P < 0.05. (**C**) Immunofluorescence of SQSTM1 in liver cryosections from mice with indicated genotypes. DAPI (blue). Scale bars, 10 μm (insets, 5 μm). Quantification of puncta per cell (means ± SEM, *N* = 3 mice per genotype). Student’s unpaired *t* test: **P < 0.005. (**D** to **F**) Western blot of the indicated proteins from the liver of fed or starved (24 hours) mice, with the indicated genotypes. β-Actin used as loading control. (**G**) Mice infected with AAV8-TBG-RPS3-RFP-GFP. Fluorescence images of hepatocytes from mice kept with food ad libitum or fasted for 24 hours. DAPI (blue). Scale bars, 10 μm (insets, 2 μm). Quantification of red-only positive puncta per cell (means ± SEM. *N* = 3, *n* = 30 cells per mouse). One-way ANOVA: *P* < 0.0001; Sidak’s test: ***P < 0.0005. (**H**) Mice with the indicated genotype were infected with AAV8-TBG-RPS3-RFP-GFP. Fluorescence images of infected hepatocytes in liver sections isolated from mice kept with food ad libitum or fasted for 24 hours. DAPI (blue). Scale bars, 10 μm (insets, 2 μm). Quantification as in (G). One-way ANOVA: *P* = 0.0002; Sidak’s test: **P < 0.005.

Next, we studied the physiological relevance of our findings in the mouse liver. We observed that the expression levels of *SQSTM1* and *ZNF598* were induced after 24 hours of starvation in the liver of C57B6 mice. Notably, liver samples from conditional transgenic mice in which *Tcfeb*-3xFlag expression was driven by tamoxifen-inducible CRE recombinase (ER-CRE) (*3*) showed higher levels of *SQSTM1* and *ZNF598* transcripts compared to nontransgenic littermates (Fig. 6, A and B). Liver sections isolated from *Tcfeb*-3xFlag showed SQSTM1 puncta in the cytoplasm of hepatocytes that, in most cases, colocalized with the lysosomal marker lysosomal-associated membrane protein 1 (Lamp1; Fig. 6C and fig. S7). In contrast to our observations in cultured cells, total SQSTM1 levels were reduced in hepatocytes, as analyzed by Western blot analysis (Fig. 6D). These data suggest that SQSTM1 puncta formed upon TFEB overexpression are degraded via autophagy more efficiently in hepatocytes than in cultured cell lines.

Next, we analyzed ribosomal protein degradation in the mouse liver under fed ad libitum and starvation conditions. We were unable to assess the levels of RPS3 in liver extract by Western blot analysis, so we measured RPS2, which acts downstream of RPS3 in the chain of ubiquitination events initiated by ZNF598 (*18*). We found that 24 hours of fasting significantly lowered RPS2 protein levels in wild-type (WT) mouse liver samples compared with fed control mice (Fig. 6, E and F). Notably, no evident reduction in other organelle markers was observed. This effect was dependent on SQSTM1, as it was strongly blunted in mice lacking SQSTM1 (*Sqstm1^−/−^* mice) (Fig. 6E). Liver samples from *Tcfeb*-3xFlag mice showed reduced levels of RPS2 protein compared with fed control mice and resembled what was observed in starved mice (Fig. 6F). To monitor RPS3 degradation in vivo, we generated an adeno-associated virus (AAV) serotype 8 vector expressing RPS3-RFP-GFP under the control of the *thyroxine-binding globulin* (TBG) promoter. AAV8-TBG-RPS3-RFP-GFP was administered systemically to wild-type, *Sqstm1*^−/−^, and *Tcfeb*-3xFlag transgenic mice. After 4 weeks, liver samples were isolated from 24-hour starved and ad libitum–fed mice. Starvation markedly increased the number of red-only puncta indicating ribophagy flux activation by starvation. Conversely, *Sqstm1*^−/−^ hepatocytes showed a reduced number of red-only puncta compared with control hepatocytes, suggesting defective starvation-induced ribophagy (Fig. 6G). The ribophagy flux was instead increased in TFEB-overexpressing mice compared with control mice (Fig. 6H). These gain- and loss-of-function data demonstrate that the ribophagy flux is regulated by TFEB via SQSTM1 regulation in the liver.

## DISCUSSION

Autophagy has long been regarded primarily as a cellular process regulated at the posttranslational level. However, about 15 years ago, two studies challenged this view by demonstrating that transcription factors belonging to the forkhead box O (FOXO) and MiT/TFE families are activated during starvation and promote the expression of genes involved in cellular catabolism, including those related to lysosomes and autophagy (*2*, *3*, *32*, *33*). The concurrent activation of FOXO and MiT/TFE and posttranscriptional autophagy regulators during starvation clearly indicated an integrated network of regulatory mechanisms that together coordinate autophagy induction.

However, a puzzling observation has been that TFEB activation can simultaneously induce autophagy and enhance mTORC1 signaling (*3*, *34*), even though mTORC1 is generally considered an autophagy suppressor. This paradox is mirrored in cancers characterized by constitutive TFEB and TFE3 activation, where elevated mTORC1 activity coexists with increased autophagy (*8*, *10*, *34*).

In this study, we demonstrate that TFEB promotes autophagy—at least in part—through activation of the SQSTM1-mediated aggrephagy pathway. This mechanism is independent of mTORC1 but relies on the local clustering of autophagy regulators within SQSTM1-positive bodies. We found that this pathway contributes to starvation-induced autophagy. Thus, our findings not only reveal an additional mechanism by which starvation induces autophagy but also provide a resolution to the apparent paradox of autophagy induction concurrent with mTORC1 activation. Furthermore, these insights may deepen our understanding of the pathogenic mechanisms underlying TFEB/TFE3–driven malignancies.

We asked which substrates were preferentially recognized by SQSTM1 upon TFEB activation. Previous studies demonstrated that various cellular cargos could accumulate in the SQSTM1 condensates, including translation initiation factors (*35*), aggregation-prone proteins (*30*), Vault complexes (*36*), and negative regulators of nuclear factor erythroid 2–related factor 2 (NRF2) signaling (*37*), among others. Regardless of the nature of the substrates, their ubiquitination appears to be a prerequisite for their sequestration by SQSTM1. In this work, we demonstrated that the enrichment of ribosomal proteins in SQSTM1 bodies can be explained, at least partly, by the ability of TFEB to induce ZNF598. ZNF598 ubiquitinates stalled ribosomes, initiating ribosomal quality control, a multistep process involving ribosome ubiquitination by ZNF598 itself and other E3 ligases, such as RING finger protein 138 (RNF138) (*38*), RNF123 (*39*), and RNF10 (*40*). Biochemical analysis of collided ribosomes showed that ZNF598 can promote formation of K63-linked polyubiquitin chains (*41*, *42*), which is often associated with degradation via the autophagy pathway. Nutrient starvation favors ribosome stalling (*19*); thus, the transcriptional induction of *ZNF598* by TFEB can be instrumental in promoting ribosome ubiquitination and autophagic degradation of ribosomes. Consistent with this, recent findings have shown that ZNF598 is a rate-limiting factor in ribosome quality control and that increasing its levels alone is sufficient to enhance this process (*43*).

Recent evidence highlights starvation-mediated ubiquitination as a critical mechanism promoting selective autophagy pathways such as ER-phagy. Notably, the ER-phagy receptor *FAM134B* is transcriptionally up-regulated by TFEB and ubiquitinated by the E3 ubiquitin ligase autocrine motility factor receptor (AMFR) during nutrient deprivation (*26*, *44*). These observations underscore that multiple, distinct autophagy induction mechanisms coexist and cooperate during starvation, not only to enhance the overall autophagic efficiency but also to direct the preferential degradation of specific cellular components. Given that the anabolic functions of the endoplasmic reticulum and ribosomes are largely dispensable under nutrient deprivation, it is plausible that cells have evolved dedicated pathways to preferentially target these organelles for degradation. Comprehensive in vivo studies examining autophagic responses in the context of tissue-specific cellular compositions will be instrumental in elucidating the regulatory principles that govern substrate specificity and coordination during starvation-induced autophagy.

## MATERIALS AND METHODS

### Cell culture and transfections

U2OS and HeLa cells were cultured in high-glucose Dulbecco’s modified Eagle’s medium (Euroclone), supplemented with 10% fetal bovine serum (Euroclone) and 1% penicillin/streptomycin. For starvation experiments, cells were synchronized by incubation in HBSS for 1 hour followed by 2 hours in complete Dulbecco’s modified Eagle’s medium and subsequently cultured in HBSS medium (Euroclone) for the indicated times. For transfection experiments, cells were transfected using Lipofectamine 3000 (Invitrogen) following a reverse transfection protocol according to the manufacturer’s instructions. Small interfering RNAs targeting *SQSTM1* and *ZNF598* were purchased from Dharmacon (smartPool siGenome) and transfected to a final concentration of 100 nM using Lipofectamine RNAiMAX (Invitrogen) Transfection Reagent following a reverse transfection protocol according to the manufacturer’s instructions. Cells were harvested 24 or 48 hours after transfection. BafA1 (Sigma-Aldrich) was used at a final concentration of 200 nM for the indicated times.

### Plasmids

pCMV TFEB-GFP plasmids were previously described (*3*). The pLenti TFEB-3xFlag plasmid was a gift from Ballabio’s lab. The pmCherry DFCP1 was provided by the De Matteis Lab. Luciferase plasmids with TFEB binding sites (CLEAR), pHAGE COX8-RFP-GFP, pHAGE SKL-RFP-GFP, pHAGE RPS3-RFP-GFP, and pHAGE RPL7-RFP-GFP, were produced by infusion cloning. pHAGE RPS3-K214R-RFP-GFP was obtained using Site-Directed Mutagenesis Kits (Agilent) according to the manufacturer’s instructions.

### Immunofluorescence

Cells were fixed for 10 min in 4% paraformaldehyde (PFA) in phosphate-buffered saline (PBS) or in ice-cold MetOH and permeabilized for 45 min in blocking buffer [0.05% (w/v) saponin, 0.5% (w/v) bovine serum albumin (BSA), 50 mM NH_4_Cl, and 0.02% NaN_3_ in PBS]. Cells were incubated in a humid chamber overnight at 4°C with primary antibodies diluted 1:200 (SQSTM1, no. GP62-C, Progen; Lamp1, no. ab24170, Abcam; RPS3, no. 9538, Cell Signal Technology; HA, no. 901501, BioLegend; LC3B, no. M1523, MBL International; WIPI-2, no. ab105459, Abcam; NBR1, no. H00004077-M01, Abnova; TAX1BP1, no. HPA024432, Sigma-Aldrich). After three washes in PBS, cells were incubated for 1 hour at room temperature with Alexa Fluor–conjugated secondary antibodies (1:200), washed three additional times, and mounted in Mowiol. All confocal experiments were acquired using LSM 880 or LSM 800 confocal microscopes equipped with a 63×/1.4–numerical aperture oil objective. Quantification was performed using ImageJ and Fiji plug-ins.

### Correlative light transmission EM (CLEM)

Cells plated on gridded glass-bottom dishes (MatTek Corporation) were transiently transfected with TFEB-GFP. Cells were fixed with 4% PFA for 1 hour at room temperature and immunolabeled for SQSTM1 (no. GP62-C, Progen) and LC3B (no. M1523, MBL International). Low-magnification bright-field images were captured to locate cells relative to the grid, followed by *Z*-stack acquisition of the cell of interest using an LSM 800 confocal microscope with a 63×/1.4–numerical aperture oil objective. After fluorescence imaging, cells were fixed with a solution containing 2.5% glutaraldehyde in 0.1 M sodium cacodylate buffer (pH 7.4). After several washes in sodium cacodylate buffer, cells were postfixed in 1% osmium tetroxide (OsO_4_) and 1.5% potassium ferricyanide (K_4_[Fe(CN)_6_]) in 0.1 M sodium cacodylate buffer for 1 hour on ice, washed with distilled water, and stained en bloc with 0.5% uranyl acetate in distilled water overnight at 4°C in the dark. Samples were then rinsed in distilled water, dehydrated with increasing concentrations of ethanol, and embedded in Epoxy resin (Sigma-Aldrich). After curing for 48 hours at 60°C, ultrathin sections (70 to 90 nm thick) were collected using an ultramicrotome (UC7, Leica microsystem), stained with uranyl acetate and Sato’s lead solutions, and observed under a transmission electron microscope Talos L120C (Thermo Fisher Scientific) operating at 120 kV. Tiled images were acquired using MAPS 3.28 software (Thermo Fisher Scientific). EM images and confocal images were aligned using the ec-CLEM plug-in of ICY software.

### Pre-embedding immunogold

After the acquisition of TFEB-GFP–positive cells, samples were fixed with 2.5% glutaraldehyde in 0.1 M cacodylate, pH 7.4, for 1 hour at room temperature. Cells were quenched with 50 mM glycine and blocked for 30 min in blocking buffer (0.2% BSA, 5% goat serum, 50 mM NH_4_Cl, 0.1% saponin, 20 mM PO_4_ buffer, and 150 mM NaCl) at room temperature. Primary anti-p62 antibody and nanogold-conjugated secondary antibody (Nanoprobes) staining was performed in blocking buffer at room temperature. Cells were postfixed for 30 min in 1% glutaraldehyde, and the nanogold particles were enlarged with a gold enhancement solution (Nanoprobes) according to the manufacturer’s instructions. Cells were postfixed with OsO_4_ and processed as described for EM. Images were acquired with a Ceta charge-coupled device camera (FEI, Thermo Fisher Scientific) using Velox 3.6.0 (FEI, Thermo Fisher Scientific) on a Talos L120C transmission electron microscope (FEI, Thermo Fisher Scientific) operating at 120 kV.

### EM analysis of autophagosomes

HeLa cells were fixed with 1% glutaraldehyde prepared in 0.2 M Hepes buffer (pH 7.3) and then postfixed with a mixture of 2% osmium tetroxide and 3% potassium ferrocyanide for 30 min. Afterward, they were incubated in 0.5% uranyl acetate at 4°C overnight. The following day, the samples were dehydrated through a graded series of ethanol and acetone and then embedded in epoxy resin. Ultrathin 60-nm sections were cut using a Leica EM UC7 ultramicrotome. Electron micrographs were acquired using a FEI Tecnai 12 electron microscope (Thermo Fisher Scientific), equipped with a Veletta charge-coupled device digital camera. The number of autophagosomes was counted within a 25-μm^2^ field of view using electron micrographs acquired at the same magnification.

### Western blot analysis

Cells were washed twice with PBS and then scraped in radioimmunoprecipitation assay lysis buffer supplemented with PhosSTOP and EDTA-free protease inhibitor tablets, 1× final concentration (Roche, Indianapolis, IN), and *N*-ethylmaleimide (1 mM final concentration). Cell lysates were lysed by a freeze-and-thaw method and collected by centrifugation at 15,000 rpm for 15 min at 4°C. The supernatant fraction was used for protein concentration determination using the colorimetric bicinchoninic acid assay (BCA) protein assay kit (Pierce Chemical Co, Boston, MA). Per sample, 20 to 70 μg of total protein was diluted in sample buffer, separated by SDS–polyacrylamide gel electrophoresis (SDS-PAGE), and transferred onto polyvinylidene difluoride or nitrocellulose membranes. The membranes were blocked with 5% BSA or 5% milk in 1× tris-buffered saline with Tween 20 for 1 hour at room temperature. The membranes were incubated with primary antibodies overnight at 4°C: β-actin (no. NB600-501, Novus Biologicals, 1:2000), LC3B (no. NB100-2220, Novus Biologicals, 1:1000), SQSTM1 (no. H00008878-M01, Abnova, 1:1000), RPS2 (no. A303-794A, Bethyl, 1:1000), RPS3 (no. 9538, Cell Signal Technology, 1:1000), Flag-M2 (no. F1804, Sigma-Aldrich, 1:1000), and ZNF598 (no. ab241092, Abcam, 1:10,000). Proteins of interest were detected with a horseradish peroxidase–conjugated goat anti-mouse or anti-rabbit IgG antibody (1:1000, Vector Laboratories) and visualized with the ECL Star Enhanced Chemiluminescent Substrate (Euroclone) according to the manufacturer’s protocol. The Western blot images were acquired using the Chemidoc-lt imaging system (UVP).

### Generation of CRISPR clones

ΔCLEAR HeLa cells were obtained through CRISPR-Cas9 technology. Single guide RNA (CATGACCGCTGTCGTAATAG) was nucleofected using the Amaxa (no. VCA1003) together with Cas9-GFP protein and the exogenous donor DNA template with mutated CLEAR motifs. The transfected GFP-positive cells were fluorescence-activated cell sorting (FACS) sorted into 96-well plates to obtain single cell–derived colonies carrying the mutations. The DNA was extracted from the clones and subjected to DNA Sanger sequencing.

### Selective autophagy tandem reporter assays

U2OS and HeLa cell lines were infected with the indicated lentivirus to generate tandem reporter cell lines. Starvation was applied for 16 hours, where indicated. Cells were collected in PBS, and the fluorescence was analyzed with a BD FACSAria III or fixed in 4% PFA for 10 min to quantify the intensity of red-only puncta over total red fluorescence per cell after image acquisition. For FACS analysis, 10,000 fluorescent events (both red and green) were collected, and the red fluorescence shift was analyzed by applying a specific threshold on the basis of the untreated samples.

### qRT-PCR

Total RNA was extracted from cultured U2OS and HeLa cells using the RNeasy Mini Kit [74106 (250), Qiagen] according to the manufacturer’s protocol. One microgram of total RNA was used for reverse transcription using the QuantiTect Reverse Transcription Kit (Qiagen) according to the manufacturer’s instructions. Quantitative reverse transcription polymerase chain reaction (qRT-PCR) was performed in triplicate using Light Cycler 480 SYBER Green I Master (Roche) and analyzed by Light Cycler 480 (Roche). The Ct (cycle threshold) values were normalized to indicated housekeeping genes, and the expression of each gene was represented as 2^(−ddCt)^ relative to control. The primers are listed in table S6.

### Luciferase assay

The promoter region of human SQSTM1 was amplified by PCR from the U2OS genome and cloned into the pGL3-basic luciferase reporter plasmid (Addgene). Cells (1 × 10^4^) were cotransfected with a luciferase plasmid together with 0.5 μg of pLX304-TFEB plasmid. The luciferase assay was performed 48 hours after transfection using the Dual Luciferase Reporter Assay System (Promega) and normalized to *Renilla* luciferase.

### DiGly and whole-cell proteomics

U2OS parental and U2OS TFEB-overexpressing cells were processed as described (*45*). Briefly, cells were washed twice with ice-cold PBS, scraped in 5 ml of denaturing lysis buffer [9 M urea, 50 mM tris, pH 8, 150 mM NaCl, 1× protease inhibitor cocktail (EDTA-free; Roche), and 50 μM deubiquitinase inhibitor PR-619 (Millipore)], and frozen in liquid nitrogen. Samples were thawed and sonicated with 3 × 20-s pulses on ice. After the removal of nonsolubilized material (15,000*g*, 10 min, 15°C), the protein concentration was determined by BCA (Pierce-Thermo), and 5 mg was used for further MS sample processing. Four biological replicates of parental U2OS and TFEB-overexpressing U2OS cells were used. After reduction with 5 mM dithiothreitol and alkylation with 10 mM chloroacetamide, lysates were digested with Lys-C (5 ng/μl; Wako) for 1 hour at room temperature. Subsequent digestion of peptides with trypsin (Promega) in a 1:200 enzyme:peptide ratio was performed. Desalted and lyophilized peptides were resuspended in 1.5 ml of immunoaffinity purification (IAP) buffer (50 mM Mops, pH 7.2, 10 mM Na_2_HPO_4_, and 50 mM NaCl) and centrifuged to remove any insoluble material (2500*g*, 5 min, 4°C). Digested peptides (0.1%) were kept in 200 μl of 0.15% trifluoroacetic acid for further whole-cell proteomic analysis. The residual supernatant was incubated with an anti-diGly antibody (32 μg per IP) conjugated to protein A agarose beads (PTMScan Ub Remnant Motif Kit, Cell Signaling) for 1 hour at 4°C. Each sample underwent two sequential rounds of IP. Unbound peptides were removed by 3× washing with IAP buffer and once with PBS. Bound material was eluted 4× with 50 μl of 0.15% trifluoroacetic acid on Micro Bio-Spin columns (Bio-Rad). Input and IP samples were desalted using custom-made C18 stage-tips (C18 material-Supelco Analytical) and resuspended in 0.1% formic acid before being applied to liquid chromatography–tandem mass spectrometry (LC-MS/MS).

Samples were loaded on a 75-μm by 15-cm custom-made fused silica capillary packed with C18AQ resin (Reprosil-PUR 120, 1.9 μm, Dr. Maisch) and separated using an Easy-nLC1200 liquid chromatograph (Thermo Fisher Scientific) followed by peptide detection on a Q Exactive HF mass spectrometer (Thermo Fisher Scientific) in data-dependent acquisition. For input samples (whole-cell proteomics), peptides were separated on a 140-min acetonitrile (ACN) gradient (2.4 to 4.8% ACN gradient for 2 min, 4.8 to 24% ACN gradient for 90 min, 24 to 35.2% ACN gradient for 20 min, 35.2 to 60% ACN gradient for 10 min, and 60 to 80% ACN gradient for 5 min) at a flow rate of 250 nl/min. For diGly samples, peptides were separated on a 60-min ACN gradient (8 to 30.4% ACN gradient for 35 min, 30.4 to 48% ACN gradient for 5 min, 48 to 80% ACN gradient for 5 min, 80% ACN for 5 min, 80 to 4% ACN gradient for 5 min, and 4% ACN for 5 min) at a flow rate of 400 nl/min. Peptides were ionized using a Nanospray Flex Ion Source (Thermo Fisher Scientific) and identified in full MS/ddMS^2^ mode. For whole-cell proteomics, the top 20 most intense peaks from each full MS scan were selected for subsequent fragmentation, with a dynamic exclusion of 120 s, excluding peptides with an unassigned charge or charges of 1 or >8. For whole proteome analysis, the MS1 resolution was set to 120,000 with a scan range of 300 to 1700 mass/charge ratio (*m*/*z*) and MS2 to 15,000. Automatic gain control (AGC) target1 was set to 3 × 10^6^, and AGC target2 was set to 1 × 10^5^. For diGly IP samples, the top five most intense peaks were picked for fragmentation, with a dynamic exclusion of 40 s, excluding peptides with an unassigned charge or charges of 1, 2, or >8. The MS1 resolution was set to 60,000 with a scan range of 300 to 1750 *m*/*z*, and MS2 was set to 30,000. AGC target1 was set to 1 × 10^6^, and AGC target2 was set to 5 × 10^5^. Data collection was controlled by Tune/Xcalibur (Thermo Fisher Scientific).

Raw data files from quadruplicate samples were analyzed using MaxQuant (1.6.0.1) Andromeda search engine in reversed decoy mode on the basis of a human reference proteome (UniProt-FASTA, UP000005640, downloaded September 2017) with a false discovery rate (FDR) of 0.01 on the protein level. Digestion parameters were set to specific digestion with trypsin/p with a maximum number of two missed cleavage sites and a minimum peptide length of 7. Methionine oxidation (15.994946), N-terminal protein acetylation (42.010565), and diGly remnant (114.042927; excluded from the C terminus) were set as variable modifications and carbamidomethylation of cysteine as fixed modification. The peptide mass tolerance was set to 20 ppm (parts per million; first search) and to 4.5 ppm (main search). Label-free quantification (with a minimum ratio count set to 2), requantification, and match-between runs were selected. Identified proteins and diGly-modified peptides were analyzed by Perseus (version 1.6.14.0). Common contaminants and reverse identifications were excluded. For the whole-cell proteome, protein groups were filtered for peptide count and MS/MS count ≥2 and a valid value in at least three of four replicates per condition. All values were log_2_ transformed. Remaining missing values were replaced by a normal distribution with 0.3 width and 1.8 downshift calculated separately for each sample. For diGly-modified peptides, peptides were filtered for a localization probability of ≥0.75 and a valid value in at least three of four replicates. Similarly, remaining missing values in the diGly peptide dataset were imputed. Statistical analysis for whole-cell proteomics was performed with a two-sided, two-sample *t* test with permutation-based FDR (*q*-value <0.05). DiGly-modified peptides were additionally normalized by the protein label-free quantification intensity before statistical analysis. Statistical testing was carried out in the same way as for the whole-cell proteome. Table S1 summarizes the diGly and whole-cell proteomics results.

### Immunoprecipitation

TFEB-3xFlag U2OS cells were harvested 48 hours after pUBQ-HA nucleofection in lysis buffer [50 mM tris-HCl (pH 8.0), 120 mM NaCl, 1% (v/v) NP-40, complete protease inhibitor cocktail, and 1 μM *N*-ethylmaleimide]. Lysates were cleared by centrifugation at 14,000 rpm for 10 min. One milligram of protein was incubated overnight at 4°C with the monoclonal anti–HA-agarose antibody (Thermo Fisher Scientific, no. 26181). Beads were then washed three times in lysis buffer and twice in 50 mM tris-HCl (pH 8.0) and 120 mM NaCl. Proteins were eluted by boiling in Laemmli buffer and separated by SDS-PAGE for Western blot analysis or processed for MS analysis. Control and TFEB-3xFlag were harvested 48 hours after plating and lysed in lysis buffer. The supernatants obtained after centrifugation were incubated overnight at 4°C with 1 μg of SQSTM1 antibody (no. H00008878-M01, Abnova, 1:1000) per 100 μg of the cell lysate after preclearing. The immunocomplexes were captured using Protein G Sepharose 4 Fast Flow (GE Healthcare, 17-0618-01) blocked beads. After 2 hours at 4°C on a rotator, the beads were then washed three times in lysis buffer, resuspended in Laemmli buffer, and separated by SDS-PAGE. Detection was performed using the anti-SQSTM1 antibody (no. GP62-C Progen) and anti–mono- and poly-ubiquitin antibodies.

### LC-MS/MS analysis

UBQ-HA-MS experiments were performed using the StageTip (iST) protocol and executed in a labeling-free setting (*46*). Samples were analyzed by LC-MS/MS using an Orbitrap Q Exactive HF mass spectrometer coupled with the NanoLC 1200 LC system. The peptides were eluted onto a homemade capillary column (75-μm inside diameter, 8-μm tip, 250-mm bed packed with Reprosil-PUR, C18-AQ, 1.9-μm particle size, 120-Å pore size) and separated at a constant flow rate of 300 nl/min according to their hydrophobicity. A binary buffer system consisting of 0.1% formic acid solution A, 80% ACN, and 0.1% formic acid solution B was used. MS data were acquired in both dependent [data-dependent acquisition (DDA)] and independent [data-independent acquisition (DIA)] modalities. Briefly, for DDA acquisition, a linear gradient of 75 min was applied. The MS spectra were obtained with the AGC target equal to 1 × 10^5^, a maximal injection time of 75 ms, and a resolution for MS/MS spectra of 30,000 at 200 *m*/*z*. The top 15 precursors were selected and fragmented by higher-energy collisional dissociation. Concerning DIA modality, a 60-min linear gradient was used. MS data acquisition was performed applying 32 variable windows, able to cover a mass range of 300 to 1650 *m*/*z*. Resolutions were set to 60,000 for MS1 and 30,000 for MS2. The AGC was 3 × 10^6^ in both MS1 and MS2, with maximum injection times of 60 ms in MS1 and 54 ms in MS2.

### Quantification and statistical elaboration (UBQ-HA interactome)

Raw data files were uploaded in MaxQuant (1.6.2.10) and Spectronaut (18.3.23) and run in a library-free search to determine protein identity and quantity. Spectra were investigated against UniProt *Homo sapiens* (UP000005640), with the following search parameters: methionine oxidation (M) and N-terminal modification of acetyl (protein N-term) as dynamic modifications and carbamidomethyl at cysteine residues (C) as a static modification. The peptides were required to be at least seven amino acids long because of the digestion with trypsin and Lys-C enzymes, counting a maximum of two missed cleavages. Both DDA and DIA data were filtered at 1% FDR on the protein level, and protein quantities were exported, followed by analysis with different software packages (Excel, Rstudio, and Perseus). Specifically, a first level of data filtering was applied to exclude contaminant proteins/peptides, reverse entries, and the identification only by a modified peptide. The signal/noise values were normalized using log_2_ transformation, while the protein abundances were grouped according to experimental conditions and filtered to achieve a minimum valid number equal to 3 in at least one group. Missing values have been replaced by random numbers that are drawn from a normal distribution. Differences between two groups were inspected using an unpaired Student’s *t* test, and feature changes with a *P* value <0.05 and a fold change (FC) ≥1 were considered significant. Functional analysis was carried out on the candidate interactors, considering the GO cellular component and Kyoto Encyclopedia of Genes and Genomes pathways as the main interesting terms. A threshold of FDR < 0.1 was applied to determine the most interesting processes. Table S4 summarizes the results of the KEGG pathways.

### Mice

The *TFEB+;Ind-Cre* mice were from Settembre *et al.* (*3*). The *SQSTM1* mouse strain was from Komatsu *et al.* (*47*). Mice were housed under specific pathogen–free conditions at 22°C and with 12-hour dark/12-hour light cycles (light cycle from 8:00 a.m. to 8:00 p.m.). Mice were fed with a standard chow diet and were of pure C57BL/6 background. All mice were observed weekly by trained personnel. All studies on mice were conducted in strict accordance with the institutional guidelines for animal research and approved by the Italian Ministry of Health, Department of Public Health, Animal Health, Nutrition, and Food Safety, in accordance with the law on animal experimentation (D.Lgs. 26/2014). Furthermore, all animal treatments were reviewed and approved in advance by the Ethics Committee at the Telethon Institute of Genetics and Medicine (TIGEM) Institute (Pozzuoli, Italy). The investigators were not blinded to allocation during experiments and outcome assessment.

### Animal procedures

For fasting experiments, mice were separated in clean cages without access to food from 2:00 p.m. to 2:00 p.m. the following day. For induction of *TFEB* expression under the control of tamoxifen-inducible Cre recombinase, mice were injected intraperitoneally with tamoxifen (100 mg/kg in corn oil) three times a week. Livers were analyzed 3 weeks after tamoxifen injection. For the AAV injection, following anesthesia with isoflurane, 1 × 10^13^ viral particles in 150 μl of PBS were injected into the retro-orbital venous plexus of 6-week-old mice with a 27-gauge syringe. Mice were euthanized 4 weeks after the AAV injection. AAV8 vectors were produced by InnovaVector (www.innovavector.eu/) by triple independent transfections of human embryonic kidney 293 cells with the RFP-GFP-RPS3 insert under the control of the TBG promoter.

### Tissue immunofluorescence

Livers were fixed overnight in 4% PFA (w/v) and then cryoprotected in sucrose solutions (10% sucrose for 2 hours, 20% sucrose overnight, and 30% sucrose overday: all w/v). Specimens were embedded in optimal cutting temperature compound (OCT), and cryosections were cut at 10 μm. Sections were incubated with blocking buffer (0.3% Triton X-100, 3% BSA, and 5% fetal bovine serum in PBS) for 3 hours at room temperature, then incubated with primary antibodies (P62 PROGEN Biotechnik GP62-C, 1:500) overnight at 4°C, and lastly stained with a secondary antibody (Alexa Fluor–labeled, 1:500) for 3 hours at room temperature in blocking buffer. For AAV-RFP-GFP-RPS3 experiments, sections were only stained with 4′,6-diamidino-2-phenylindole (DAPI; 1:1000 in PBS for 30 min at room temperature) and then mounted. Images were captured using an LSM 880 confocal microscope equipped with a 63×/1.4–numerical aperture oil objective.

### Tissue Western blotting

Liver samples were lysed using a TissueLyser (Qiagen) in radioimmunoprecipitation assay buffer supplemented with 0.5% SDS, PhosSTOP, and EDTA-free protease inhibitor tablets (Roche). Samples were incubated for 30 min on ice and briefly sonicated on ice, and the soluble fraction was isolated by centrifugation at 14,000 rpm for 15 min at 4°C. BCA assay was performed to determine the protein concentration. Samples were run on SDS polyacrylamide gels and transferred onto nitrocellulose membranes at 100 V for 1 hour at 4°C. Membranes were blocked with 5% BSA for 1 hour and then incubated overnight with the following antibodies diluted 1:1000 in 5% BSA: RPS2 (Bethy, A303-79A), P62 clone 2C11 (Abnova, H00008878-M01), TFEB (Bethyl Laboratories, A303-673A), calnexin (Enzo Life Sciences, ADI-SPA 860), β-actin (Novus Biologicals, NB600-501), and citrate synthase (Abcam, ab96600).

### Functional analysis on proteomics data

Gene ontology enrichment analysis was performed on induced proteins using the DAVID Bioinformatic tool (*48*, *49*), restricting the output to cellular compartment (CC) terms. The threshold for the statistical significance of gene ontology enrichment analysis was FDR < 0.1 and enrichment score (ES) ≥1.5. The top five significant CC clusters are shown in Fig. 4, while the complete list of CC clusters is reported in table S5.

### TFEB binding in *SQSTM1* and *ZNF598* promoters

TFEB binding at the *ZNF598* and *SQSTM1 loci* was examined using data from the ChIP-seq experiment performed in (*27*). The UCSC genome browser was used to verify the binding sites and to isolate the specific binding coordinates on the reference genome Hg19.

### Statistical analysis

Results are presented as bar graphs indicating the standard error of the mean (SEM). Statistical analysis was performed using an unpaired two-tailed Student’s *t* test, one-way analysis of variance (ANOVA), or two-way ANOVA and post hoc test with Sidak’s multiple comparison test where required. *P* values <0.05 were considered statistically significant. All experiments were repeated at least three times. The exact sample size for each experiment is reported as *N* in the caption of each figure.

## Supplementary Materials

### The PDF file includes:

Figs. S1 to S7 Legends for tables S1 to S6 References
