# Supplementary material for: TFEB coordinates autophagosome biogenesis and ribophagy during starvation via SQSTM1
Source: Sci Adv. 2026 Jan 1;12(1):eaea9302. doi: 10.1126/sciadv.aea9302 (PMC12757066; doi:10.1126/sciadv.aea9302)
Supplement: Supplementary file 1 — Figs. S1 to S7 Legends for tables S1 to S6 References [file sciadv.aea9302_sm.pdf]

Science Advances
AAAS

Supplementary Materials for

TFEB coordinates autophagosome biogenesis and ribophagy during starvation via SQSTM1

Maria Iavazzo *et al.*

Corresponding author: Carmine Settembre, settembre@tigem.it

Sci. Adv. **12**, eaea9302 (2026)
DOI: 10.1126/sciadv.aea9302

The PDF file includes:

Figs. S1 to S7
Legends for tables S1 to S6
References

Other Supplementary Material for this manuscript includes the following:

Tables S1 to S6

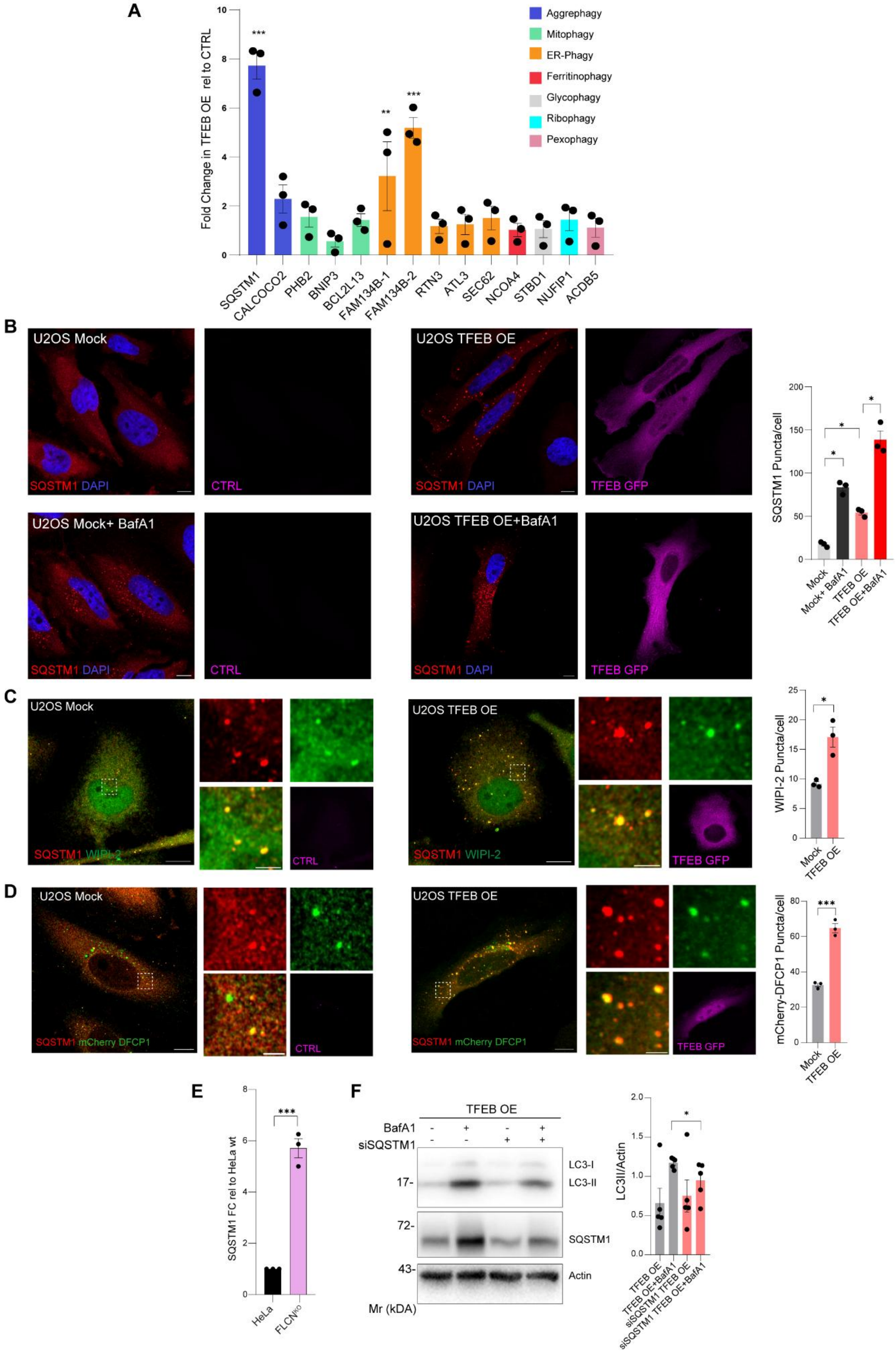
A
Fold Change in TFEB OE rel to CTRL
Aggrephagy
Mitophagy
ER-Phagy
Ferritinophagy
Glycophagy
Ribophagy
Pexophagy
SQSTM1
CALCOCO2
PHB2
BNIP3
BCL2L13
FAM134B-1
FAM134B-2
RTN3
ATL3
SEC62
NCOA4
STBD1
NUFIP1
ACDB5
B
U2OS Mock
SQSTM1 DAPI
CTRL
U2OS TFEB OE
TFEB GFP
U2OS Mock+ BafA1
U2OS TFEB OE+BafA1
SQSTM1 Puncta/cell
Mock
Mock+ BafA1
TFEB OE
TFEB OE+BafA1
C
SQSTM1 WIPI-2
WIPI-2 Puncta/cell
D
SQSTM1 mCherry DFCP1
mCherry-DFCP1 Puncta/cell
E
SQSTM1 FC rel to HeLa wt
HeLa
FLCN KO
F
TFEB OE
BafA1
siSQSTM1
LC3-I
LC3-II
SQSTM1
Actin
Mr (kDA)
LC3II/Actin
TFEB OE+BafA1
siSQSTM1 TFEB OE
siSQSTM1 TFEB OE+BafA1

Fig. S1: (**A**) qRT–PCR analysis of indicated autophagy receptors in TFEB-3xFlag (TFEB OE) U2OS cells. Fold change normalized to *cyclophilin* gene expression and relative to CTRL (mean ± SEM of $N = 3$). Two-way ANOVA. Sidak's test, $^{**}p < 0.005$, $^{***}p < 0.0001$. (**B**) Immunofluorescence staining of SQSTM1 (red) in mock-transfected and TFEB-GFP-transfected (purple) cells. BafA1 was added where indicated (BafA1 200 nM, 2 hours). Scale bars, 10 µm. The graph shows quantification of SQSTM1 puncta/cell. Mean ± SEM of $N = 3$, $n = 30$ cells/treatment. One-way ANOVA $^{**}P = 0.0015$. Sidak's multiple comparisons test, $^{*}p < 0.05$. (**C**) Co-immunofluorescence of SQSTM1 (red) and WIPI-2 (green) in mock-transfected and TFEB-GFP-transfected (purple) cells. Scale bars, 10 µm (insets, 2 µm). Quantification of WIPI-2 puncta/cell (mean ± SEM of $N = 3$, $n = 25$ cells/sample). Student's unpaired t-test, $^{*}p < 0.05$. (**D**) Immunofluorescence of SQSTM1 (red) and mCherry-DFCP1 (green) in mock-transfected and TFEB-GFP-transfected (purple) cells. Scale bars, 10 µm (insets, 2 µm). Quantification of mCherry-DFCP1 puncta/cell. Mean ± SEM of $N = 3$, $n = 29$ cells/sample. Student's unpaired t-test, $^{***}p < 0.0005$. (**E**) qRT-PCR analysis of *SQSTM1* in HeLa and $FLCN^{KO}$ HeLa cells. Fold change normalized to *Actin* and expressed relative to WT. Mean ± SEM of $N = 3$. Student's unpaired t-test, $^{***}p < 0.0005$. (**F**) Western blot analysis of SQSTM1 and LC3B proteins in U2OS expressing TFEB-3xFlag, ± SQSTM1 silencing (siSQSTM1); BafA1 treatment was performed where indicated (200 nM, 4 hours). β-actin was used as a loading control. Quantification of LC3BII relative to actin. Mean ± SEM, $N = 5$. Student's unpaired t-test (one-tailed), $^{*}p < 0.04$.

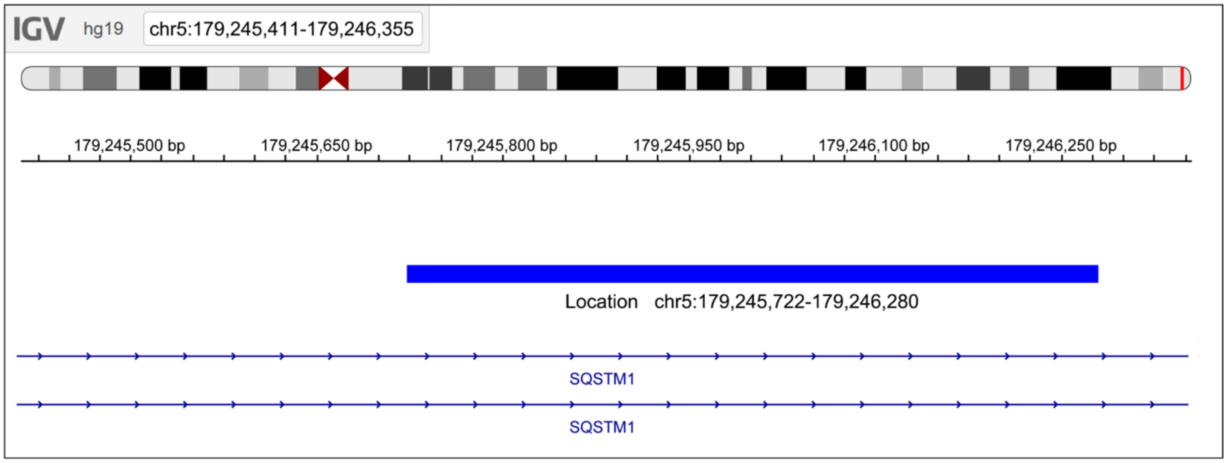

Fig. S2: IGV visualization in De Cegli *et al*. 2022 (*50*), of TFEB binding sites in the SQSTM1 *locus*, as defined by ChIPseq analysis performed in Gambardella *et al*., 2020 (*27*). The binding site is located in the promoter region of SQSTM1 at -1562 nucleotides from the transcription start site

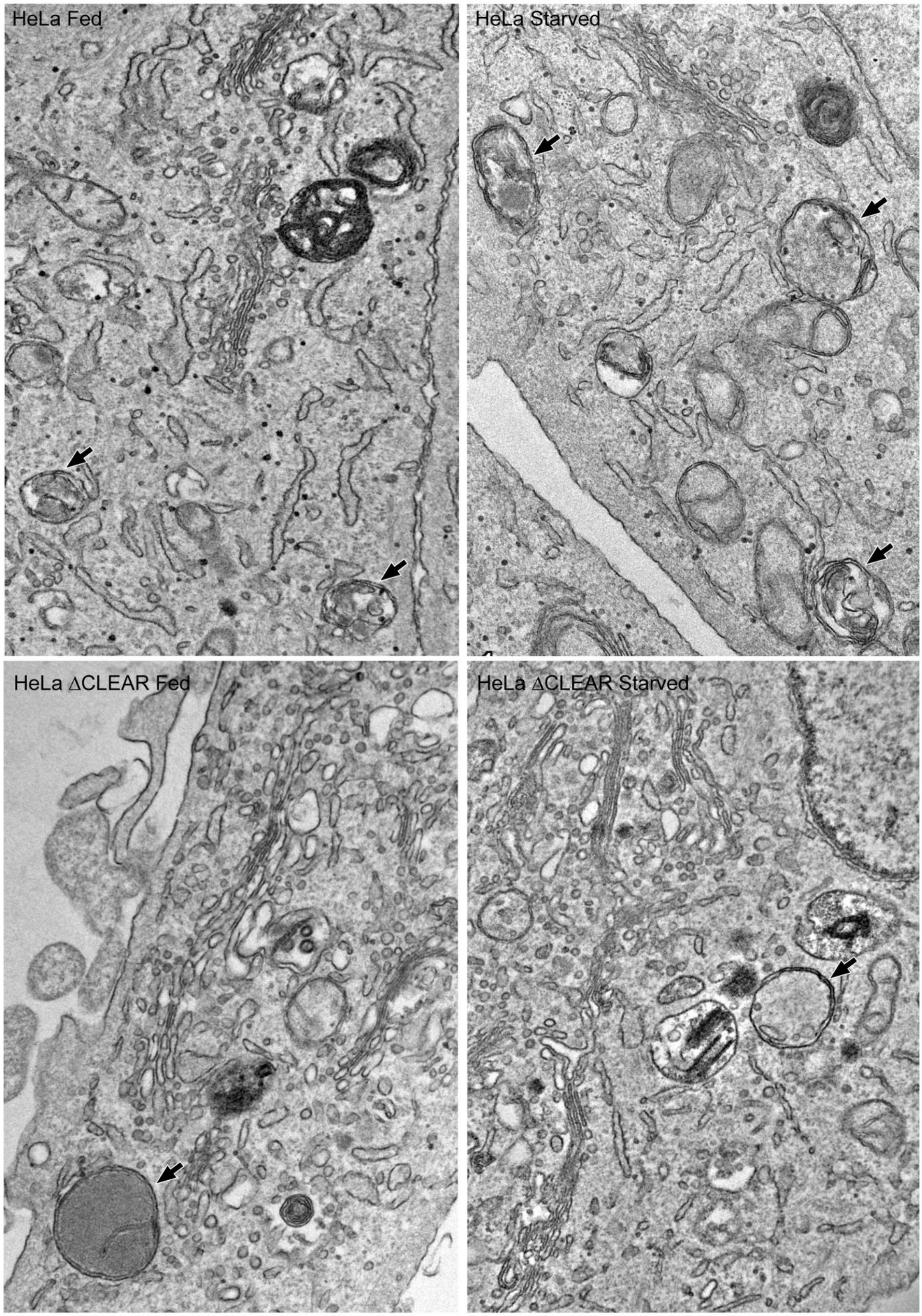
HeLa Fed
HeLa Starved
HeLa ΔCLEAR Fed
HeLa ΔCLEAR Starved
500 nm

Fig. S3: TEM analysis of autophagosomes in HeLa and HeLa ΔCLEAR ± HBSS (2 hours). Arrows indicate the autophagosomes. Scale bar, 500 nm.

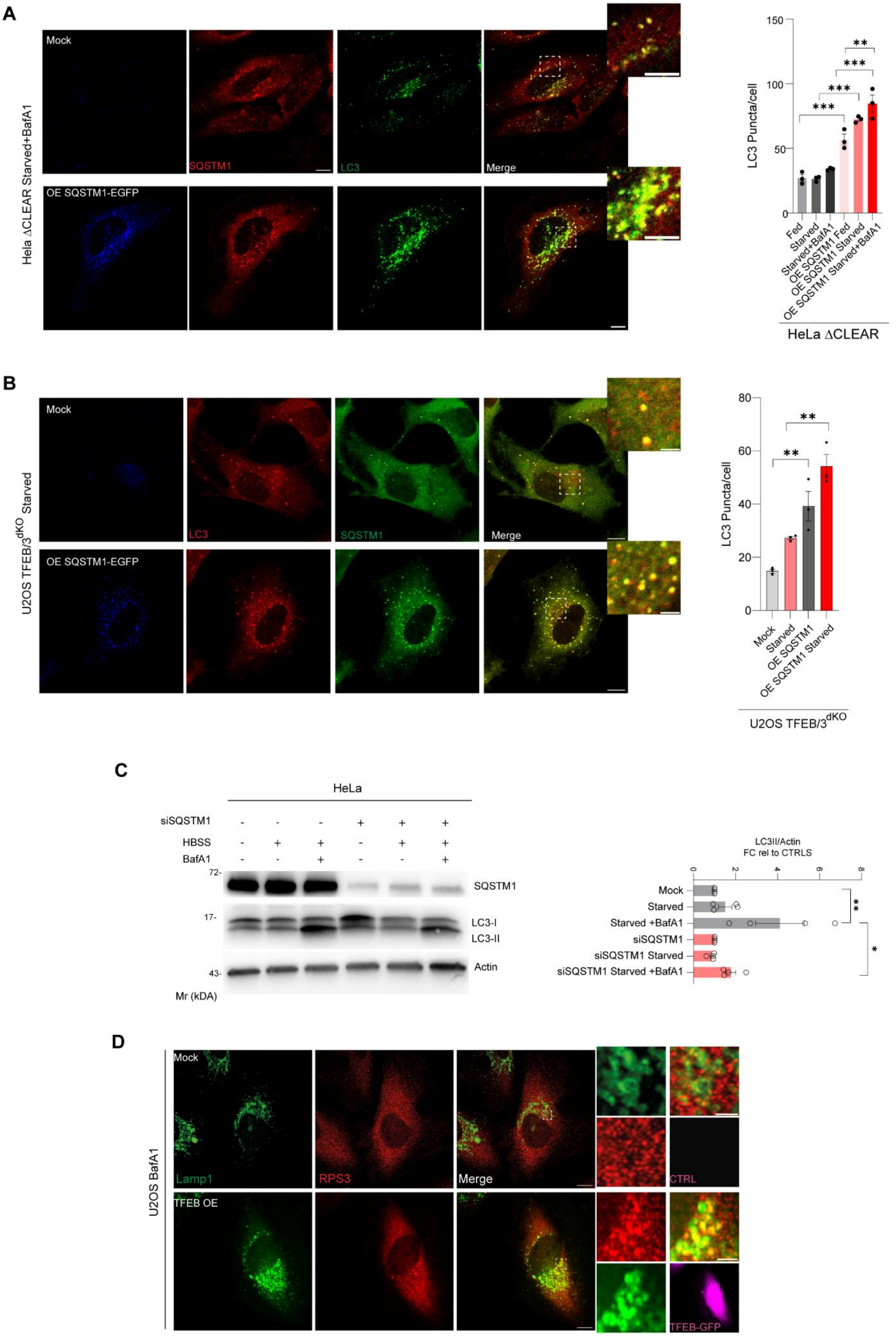
A
Hela ΔCLEAR Starved+BafA1
Mock
SQSTM1
LC3
Merge
OE SQSTM1-EGFP
LC3 Puncta/cell
150
100
50
0
**
***
***
***
Fed
Starved
Starved+BafA1
OE SQSTM1 Fed
OE SQSTM1 Starved
OE SQSTM1 Starved+BafA1
HeLa ΔCLEAR
B
U2OS TFEB/3dKO Starved
Mock
LC3
SQSTM1
Merge
OE SQSTM1-EGFP
LC3 Puncta/cell
80
60
40
20
0
**
**
Mock
Starved
OE SQSTM1
OE SQSTM1 Starved
U2OS TFEB/3dKO
C
HeLa
siSQSTM1 - - - + + +
HBSS - + + - + +
BafA1 - - + - - +
72-
17-
43-
Mr (kDA)
SQSTM1
LC3-I
LC3-II
Actin
LC3II/Actin
FC rel to CTRLS
0
2
4
6
8
Mock
Starved
Starved +BafA1
siSQSTM1
siSQSTM1 Starved
siSQSTM1 Starved +BafA1
**
*
D
U2OS BafA1
Mock
Lamp1
RPS3
Merge
CTRL
TFEB OE
TFEB-GFP

Fig. S4: (**A**) Co-immunofluorescence of SQSTM1 (red) and LC3B (green) in mock-transfected and SQSTM1-EGFP-transfected (blue) ΔCLEAR HeLa cells. Scale bars, 10 µm (insets, 5 µm). Quantification of LC3B puncta/cell. Mean ± SEM of $N = 3$, $n = 30$ cells/condition. One-way ANOVA, $P < 0.0001$. Sidak's test, ***$p < 0.0005$, *$p < 0.05$. (**B**) Co-immunofluorescence of SQSTM1 (green) and LC3B (red) in mock and SQSTM1-EGFP-transfected (blue) TFEB/3^{dKO} U2OS cells. Scale bars, 10 µm (insets, 2 µm). Quantification of LC3B puncta/cell. Mean ± SEM of $N = 3$, $n = 24$ cells/condition. One-way ANOVA, *** $P = 0.0003$. Sidak's test, **$p < 0.005$. (**C**) Western blot of SQSTM1 and LC3B proteins in HeLa cells, either scrambled or siSQSTM1 transfected, ± HBSS (4 hours) or BafA1 treatment (200 nM, 4 hours). β-actin was used as a loading control. Quantification of LC3BII normalized to β-actin and relative to mock. Mean ± SEM of $N = 4$. One-way ANOVA, ***$P = 0.0021$. Sidak's test **p < 0.005; *p < 0.05. (**D**) Co-immunofluorescence staining of Lamp1 (green) and RPS3 (red) in mock-transfected and TFEB-GFP-transfected (blue) U2OS. Scale bars, 10 µm.

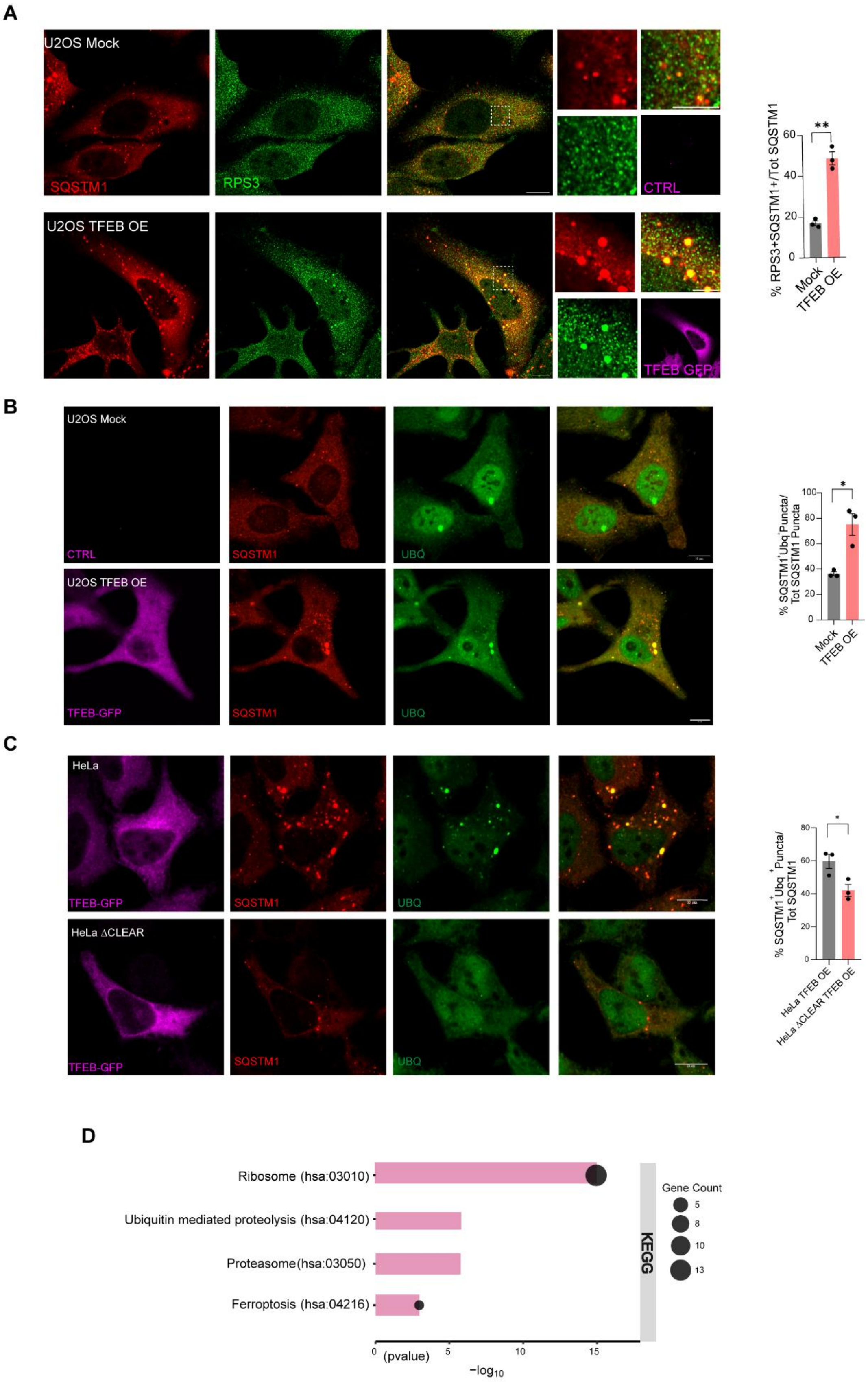
A
U2OS Mock
SQSTM1
RPS3
CTRL
U2OS TFEB OE
TFEB GFP
% RPS3+SQSTM1+/Tot SQSTM1
Mock
TFEB OE
B
U2OS Mock
CTRL
SQSTM1
UBQ
U2OS TFEB OE
TFEB-GFP
Mock
TFEB OE
C
HeLa
HeLa ΔCLEAR
TFEB-GFP
SQSTM1
UBQ
HeLa TFEB OE
HeLa ΔCLEAR TFEB OE
D
Ribosome (hsa:03010)
Ubiquitin mediated proteolysis (hsa:04120)
Proteasome(hsa:03050)
Ferroptosis (hsa:04216)
KEGG
Gene Count
5
8
10
13
(pvalue)
−log10

Fig. S5: (**A**) Co-immunofluorescence of SQSTM1 (red) and RPS3 (green) in mock-transfected and TFEB-GFP overexpressing U2OS. Scale bars, 10 µm (insets, 5 µm). Quantification of the percentage of $RPS3^{+}SQSTM1^{+}$ relative to total SQSTM1 puncta/cell. Mean ± SEM of $N = 3$, $n = 30$ cells/genotype. Student's unpaired t-test, $^{**}p < 0.005$. (**B**) Co-immunofluorescence of SQSTM1 (red) and Ubiquitin (green) in mock-transfected and in TFEB-GFP (purple) overexpressing U2OS. Scale bars, 10 µm. Quantification of the percentage of $Ubq^{+}/SQSTM1^{+}$ relative to total SQSTM1 puncta/cell. Mean ± SEM of $N = 3$, $n = 29$ cells/sample. Student's unpaired t-test, $^{*}p < 0.05$. (**C**) Co-immunofluorescence of SQSTM1 (red), Ubiquitin (green), and TFEB-GFP (purple) in WT and ΔCLEAR HeLa cells overexpressing TFEB-GFP (purple). Scale bars, 10 µm. Quantification of the percentage of $Ubq^{+}/SQSTM1^{+}$ relative to total SQSTM1 puncta/cell. Mean ± SEM of $N = 3$, $n = 31$ cells/condition. Student's unpaired t-test, $^{*}p < 0.05$. (**D**) Bar chart of the UBQ-HA (see Fig. 5A) interactome genes enriched in the KEGG database (top terms, Table S4).

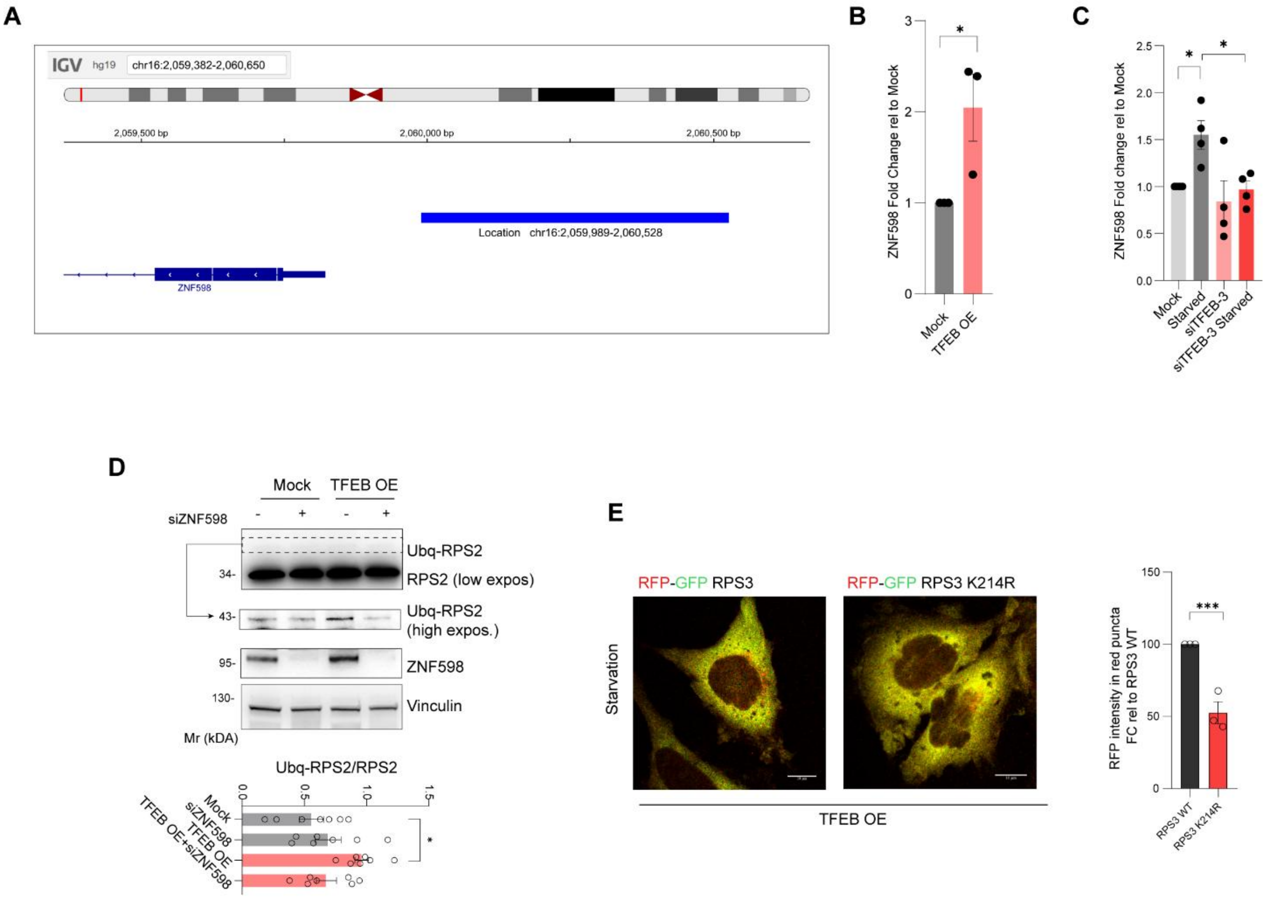

Fig. S6: (**A**) IGV visualization in De Cegli *et al.* 2022 (*50*), of TFEB binding sites in the ZNF598 *locus*, as defined by ChIPseq analysis performed in Gambardella *et al*., 2020 (*27*). The binding site is in the promoter region of ZNF598 at -167 nucleotides from the transcription start site. (**B**) qRT–PCR of *ZNF598* in mock-transfected and overexpressing TFEB-GFP HeLa. Fold change normalized to *Actin* and relative to mock-transfected cells. Mean ± SEM of $N = 3$ biological replicates. Student's unpaired t-test, $*p < 0.05$. (**C**) qRT–PCR analysis of *ZNF598* in HeLa, either scramble-silenced (mock) or si-TFEB-TFE3 ± HBSS (4 hours). Fold change relative to mock and normalized to *HPRT*. Mean ± standard SEM of $N = 4$. One-way ANOVA, $*P = 0.01$. Sidak's test. $*p < 0.05$. (**D**) Western blot of RPS2 and ZNF598 proteins in mock-transfected and TFEB-overexpressing U2OS, without or with siZNF598. Vinculin was used as a loading control. The dotted box and arrow indicate the region of the blot corresponding to ubiquitinated RPS2 that required high exposure (expos). Quantification of the Ubq-RPS2 relative to total RPS2. $N = 7$, mean ±

SEM. One-way ANOVA, $P = 0.02$. Sidak's test, $*p < 0.012$. (**E**) Fluorescence images of RPS3 WT and K214R tandem reporter in TFEB overexpressing cells starved in HBSS (16 hours). Scale bars, 10 µm. Quantification of RFP puncta intensity/cell. Mean ± SEM of $N = 3$, $n = 28$ cells/condition. Student's unpaired t-test, $***p < 0.0005$.

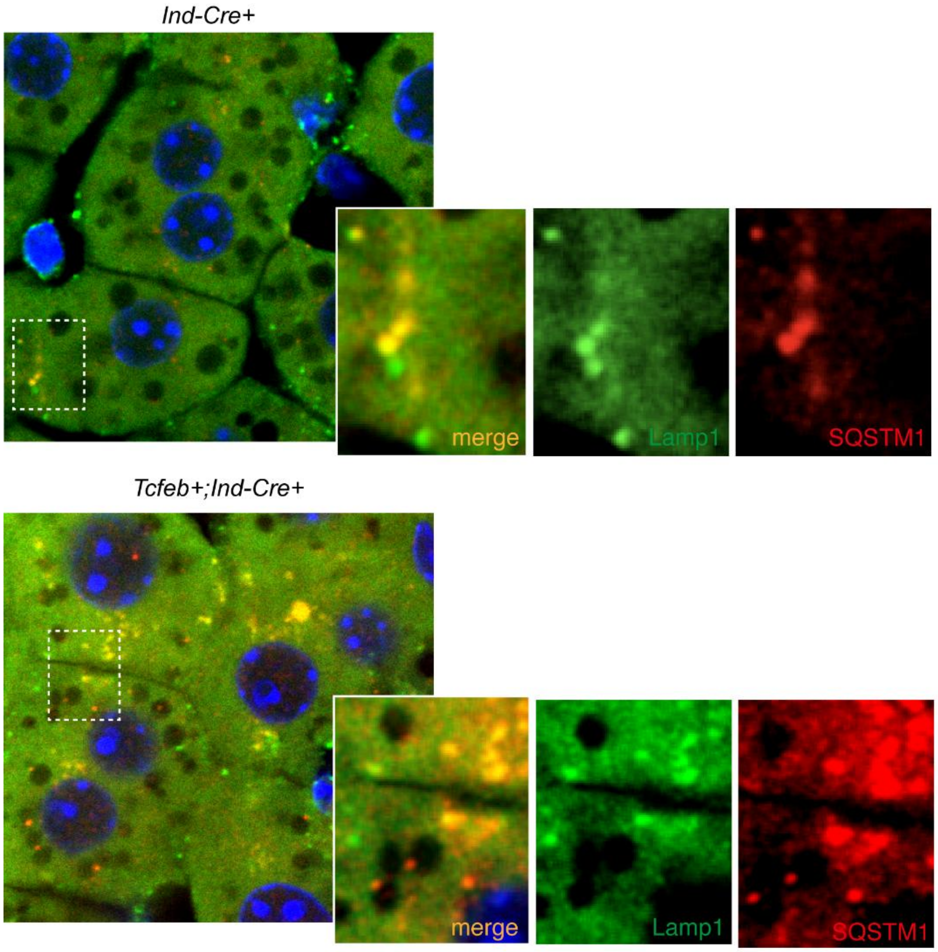

Fig. S7: Immunofluorescence of Lamp1 (green) and SQSTM1 (red) in liver cryosections from *wt;Ind-Cre+* and *Tcfeb;Ind-Cre+* mice. DAPI (blue). Scale bars, 10 µm.

Other Supplementary Materials:

Tables S1 Whole-cell proteome and ubiquitinated peptides identified by diGly proteomics in TFEB-3xFlag and WT U2OS cells.

Tables S2 UBQ-HA interactome in TFEB-3xFlag U2OS.

Tables S3 UBQ-HA interactome in TFEB 3×Flag U2OS cells transfected with HA-tagged ubiquitin, either without (UBQ-HA) or with ZNF598 silencing (UBQ-HA + siZNF598).

Tables S4 Gene Ontology enrichment analysis for Cellular Component in the KEGG database of genes enriched in the UBQ-HA interactome in TFEB-3xFlag U2OS.

Table S5 Gene Ontology Enrichment Analysis on Cellular Component (CC) on 465 positively enriched proteins. All significant CC (FDR<10% and Enrichment Score >1.5) in which the induced proteins are mainly localized.

Tables S6 List of qRT-PCR primers used in this study.

REFERENCES

1. C. Settembre, R. M. Perera, Lysosomes as coordinators of cellular catabolism, metabolic signalling and organ physiology. *Nat. Rev. Mol. Cell Biol.* **25**, 223–245 (2024).

2. M. Sardiello, M. Palmieri, A. di Ronza, D. L. Medina, M. Valenza, V. A. Gennarino, C. Di Malta, F. Donaudy, V. Embrione, R. S. Polishchuk, S. Banfi, G. Parenti, E. Cattaneo, A. Ballabio, A gene network regulating lysosomal biogenesis and function. *Science* **325**, 473–477 (2009).

3. C. Settembre, C. Di Malta, V. A. Polito, M. Garcia Arencibia, F. Vetrini, S. Erdin, S. U. Erdin, T. Huynh, D. Medina, P. Colella, M. Sardiello, D. C. Rubinsztein, A. Ballabio, TFEB links autophagy to lysosomal biogenesis. *Science* **332**, 1429–1433 (2011).

4. C. Settembre, R. De Cegli, G. Mansueto, P. K. Saha, F. Vetrini, O. Visvikis, T. Huynh, A. Carissimo, D. Palmer, T. J. Klisch, A. C. Wollenberg, D. Di Bernardo, L. Chan, J. E. Irazoqui, A. Ballabio, TFEB controls cellular lipid metabolism through a starvation-induced autoregulatory loop. *Nat. Cell Biol.* **15**, 647–658 (2013).

5. C. Settembre, R. Zoncu, D. L. Medina, F. Vetrini, S. Erdin, S. Erdin, T. Huynh, M. Ferron, G. Karsenty, M. C. Vellard, V. Facchinetti, D. M. Sabatini, A. Ballabio, A lysosome-to-nucleus signalling mechanism senses and regulates the lysosome via mTOR and TFEB. *EMBO J.* **31**, 1095–1108 (2012).

6. A. Roczniak-Ferguson, C. S. Petit, F. Froehlich, S. Qian, J. Ky, B. Angarola, T. C. Walther, S. M. Ferguson, The transcription factor TFEB links mTORC1 signaling to transcriptional control of lysosome homeostasis. *Sci. Signal.* **5**, ra42 (2012).

7. J. A. Martina, Y. Chen, M. Gucek, R. Puertollano, MTORC1 functions as a transcriptional regulator of autophagy by preventing nuclear transport of TFEB. *Autophagy* **8**, 903–914 (2012).

8. R. M. Perera, S. Stoykova, B. N. Nicolay, K. N. Ross, J. Fitamant, M. Boukhali, J. Lengrand, V. Deshpande, M. K. Selig, C. R. Ferrone, J. Settleman, G. Stephanopoulos, N. J. Dyson, R. Zoncu,

S. Ramaswamy, W. Haas, N. Bardeesy, Transcriptional control of autophagy–lysosome function drives pancreatic cancer metabolism. *Nature* **524**, 361–365 (2015).

9. A. Calcagnì, L. Kors, E. Verschuren, R. De Cegli, N. Zampelli, E. Nusco, S. Confalonieri, G. Bertalot, S. Pece, C. Settembre, G. G. Malouf, J. C. Leemans, E. De Heer, M. Salvatore, D. J. Peters, P. P. Di Fiore, A. Ballabio, Modelling TFE renal cell carcinoma in mice reveals a critical role of WNT signaling. *eLife*, e17047 **5** (2016).

10. G. Napolitano, C. Di Malta, A. Esposito, M. E. G. De Araujo, S. Pece, G. Bertalot, M. Matarese, V. Benedetti, A. Zampelli, T. Stasyk, D. Siciliano, A. Venuta, M. Cesana, C. Vilardo, E. Nusco, J. Monfregola, A. Calcagnì, P. P. Di Fiore, L. A. Huber, A. Ballabio, A substrate-specific mTORC1 pathway underlies Birt–Hogg–Dubé syndrome. *Nature* **585**, 597–602 (2020).

11. S. Martens, C. Behrends, Molecular mechanisms of selective autophagy. *J. Mol. Biol.* **432**, 1–2 (2020).

12. B. Bauer, S. Martens, L. Ferrari, Aggrephagy at a glance. *J. Cell Sci.* **136**, jcs260888 (2023).

13. R. Kurusu, H. Morishita, M. Komatsu, p62 bodies: Cytosolic zoning by phase separation. *J. Biochem.* **175**, 141–146 (2024).

14. B. Bauer, J. Idinger, M. Schuschnig, L. Ferrari, S. Martens, Recruitment of autophagy initiator TAX1BP1 advances aggrephagy from cargo collection to sequestration. *EMBO J.* **43**, 5910–5940 (2024).

15. R. B. Damgaard, The ubiquitin system: From cell signalling to disease biology and new therapeutic opportunities. *Cell Death Differ.* **28**, 423–426 (2021).

16. G. Zaffagnini, A. Savova, A. Danieli, J. Romanov, S. Tremel, M. Ebner, T. Peterbauer, M. Sztacho, R. Trapannone, A. K. Tarafder, C. Sachse, S. Martens, Phasing out the bad-how SQSTM1/p62 sequesters ubiquitinated proteins for degradation by autophagy. *Autophagy* **14**, 1280–1282 (2018).

17. S. Filbeck, F. Cerullo, S. Pfeffer, C. A. P. Joazeiro, Ribosome-associated quality-control mechanisms from bacteria to humans. *Mol. Cell* **82**, 1451–1466 (2022).

18. C. Meyer, A. Garzia, P. Morozov, H. Molina, T. Tuschl, The G3BP1-Family-USP10 deubiquitinase complex rescues ubiquitinated 40S subunits of ribosomes stalled in translation from lysosomal degradation. *Mol. Cell* **77**, 1193–1205.e5 (2020).

19. H. An, A. Ordureau, M. Körner, J. A. Paulo, J. W. Harper, Systematic quantitative analysis of ribosome inventory during nutrient stress. *Nature* **583**, 303–309 (2020).

20. G. A. Wyant, M. Abu-Remaileh, E. M. Frenkel, N. N. Laqtom, V. Dharamdasani, C. A. Lewis, S. H. Chan, I. Heinze, A. Ori, D. M. Sabatini, NUFIP1 is a ribosome receptor for starvation-induced ribophagy. *Science* **360**, 751–758 (2018).

21. Y. Chen, J. Hu, P. Zhao, J. Fang, Y. Kuang, Z. Liu, S. Dong, W. Yao, Y. Ding, X. Wang, Y. Pan, J. Wu, J. Zhao, J. Yang, Z. Xu, X. Liu, Y. Zhang, C. Wu, L. Zhang, M. Fan, S. Feng, Z. Hong, Z. Yan, H. Xia, K. Tang, B. Yang, W. Liu, Q. Sun, K. Mei, W. Zou, Y. Huang, D. Feng, C. Yi, Rpl12 is a conserved ribophagy receptor. *Nat. Cell Biol.* **27**, 477–492 (2025).

22. P. C. Monem, J. A. Arribere, A ubiquitin language communicates ribosomal distress. *Semin. Cell Dev. Biol.* **154**, 131–137 (2024).

23. C. A. P. Joazeiro, Ribosomal stalling during translation: Providing substrates for ribosome-associated protein quality control. *Annu. Rev. Cell Dev. Biol.* **33**, 343–368 (2017).

24. S. Juszkiewicz, V. Chandrasekaran, Z. Lin, S. Kraatz, V. Ramakrishnan, R. S. Hegde, ZNF598 is a quality control sensor of collided ribosomes. *Mol. Cell* **72**, 469–481.e7 (2018).

25. A. R. López, M. H. Jørgensen, J. F. Havelund, F. S. Arendrup, S. P. Kolapalli, T. M. Nielsen, E. Pais, C. J. Beese, A. Abdul-Al, A. C. Vind, J. Bartek, S. Bekker-Jensen, M. Montes, P. Galanos, N. Faergeman, L. Happonen, L. B. Frankel, Autophagy-mediated control of ribosome homeostasis in oncogene-induced senescence. *Cell Rep.* **42**, 113381 (2023).

26. L. Cinque, C. De Leonibus, M. Iavazzo, N. Krahmer, D. Intartaglia, F. G. Salierno, R. De Cegli, C. Di Malta, M. Svelto, C. Lanzara, M. Maddaluno, L. G. Wanderlingh, A. K. Huebner, M. Cesana, F. Bonn, E. Polishchuk, C. A. Hübner, I. Conte, I. Dikic, M. Mann, A. Ballabio, F. Sacco, P. Grumati, C. Settembre, MiT/TFE factors control ER-phagy via transcriptional regulation of FAM134B. *EMBO J.* **39**, e105696 (2020).

27. G. Gambardella, L. Staiano, M. N. Moretti, R. De Cegli, L. Fagnocchi, G. Di Tullio, S. Polletti, C. Braccia, A. Armirotti, A. Zippo, A. Ballabio, M. A. De Matteis, D. di Bernardo, GADD34 is a modulator of autophagy during starvation. *Sci. Adv.* **6**, eabb0205 (2020).

28. M. H. Sahani, E. Itakura, N. Mizushima, Expression of the autophagy substrate SQSTM1/p62 is restored during prolonged starvation depending on transcriptional upregulation and autophagy-derived amino acids. *Autophagy* **10**, 431–441 (2014).

29. S. Kimura, T. Noda, T. Yoshimori, Dissection of the autophagosome maturation process by a novel reporter protein, tandem fluorescent-tagged LC3. *Autophagy* **3**, 452–460 (2007).

30. G. Bjørkøy, T. Lamark, A. Brech, H. Outzen, M. Perander, A. Overvatn, H. Stenmark, T. Johansen, p62/SQSTM1 forms protein aggregates degraded by autophagy and has a protective effect on huntingtin-induced cell death. *J. Cell Biol.* **171**, 603–614 (2005).

31. A. Garzia, S. M. Jafarnejad, C. Meyer, C. Chapat, T. Gogakos, P. Morozov, M. Amiri, M. Shapiro, H. Molina, T. Tuschl, N. Sonenberg, The E3 ubiquitin ligase and RNA-binding protein ZNF598 orchestrates ribosome quality control of premature polyadenylated mRNAs. *Nat. Commun.* **8**, 16056 (2017).

32. G. Milan, V. Romanello, F. Pescatore, A. Armani, J.-H. Paik, L. Frasson, A. Seydel, J. Zhao, R. Abraham, A. L. Goldberg, B. Blaauw, R. A. DePinho, M. Sandri, Regulation of autophagy and the ubiquitin–proteasome system by the FoxO transcriptional network during muscle atrophy. *Nat. Commun.* **6**, 6670 (2015).

33. C. Mammucari, G. Milan, V. Romanello, E. Masiero, R. Rudolf, P. Del Piccolo, S. J. Burden, R. Di Lisi, C. Sandri, J. Zhao, A. L. Goldberg, S. Schiaffino, M. Sandri, FoxO3 controls autophagy in skeletal muscle in vivo. *Cell Metab.* **6**, 458–471 (2007).

34. C. Di Malta, D. Siciliano, A. Calcagni, J. Monfregola, S. Punzi, N. Pastore, A. N. Eastes, O. Davis, R. De Cegli, A. Zampelli, L. G. Di Giovannantonio, E. Nusco, N. Platt, A. Guida, M. H. Ogmundsdottir, L. Lanfrancone, R. M. Perera, R. Zoncu, P. G. Pelicci, C. Settembre, A. Ballabio, Transcriptional activation of RagD GTPase controls mTORC1 and promotes cancer growth. *Science* **356**, 1188–1192 (2017).

35. A. Danieli, G. Vucak, M. Baccarini, S. Martens, Sequestration of translation initiation factors in p62 condensates. *Cell Rep.* **42**, 113583 (2023).

36. R. Kurusu, Y. Fujimoto, H. Morishita, D. Noshiro, S. Takada, K. Yamano, H. Tanaka, R. Arai, S. Kageyama, T. Funakoshi, S. Komatsu-Hirota, H. Taka, S. Kazuno, Y. Miura, M. Koike, T. Wakai, S. Waguri, N. N. Noda, M. Komatsu, Integrated proteomics identifies p62-dependent selective autophagy of the supramolecular vault complex. *Dev. Cell* **58**, 1189–1205.e11 (2023).

37. S. Kageyama, S. R. Gudmundsson, Y.-S. Sou, Y. Ichimura, N. Tamura, S. Kazuno, T. Ueno, Y. Miura, D. Noshiro, M. Abe, T. Mizushima, N. Miura, S. Okuda, H. Motohashi, J.-A. Lee, K. Sakimura, T. Ohe, N. N. Noda, S. Waguri, E.-L. Eskelinen, M. Komatsu, p62/SQSTM1-droplet serves as a platform for autophagosome formation and anti-oxidative stress response. *Nat. Commun.* **12**, 16 (2021).

38. W. Kim, H. Youn, S. Lee, E. Kim, D. Kim, J. Sub Lee, J.-M. Lee, B. Youn, RNF138-mediated ubiquitination of rpS3 is required for resistance of glioblastoma cells to radiation-induced apoptosis. *Exp. Mol. Med.* **50**, e434 (2018).

39. Y. Jung, H. D. Kim, H. W. Yang, H. J. Kim, C.-Y. Jang, J. Kim, Modulating cellular balance of Rps3 mono-ubiquitination by both Hel2 E3 ligase and Ubp3 deubiquitinase regulates protein quality control. *Exp. Mol. Med.* **49**, e390 (2017).

40. A. Garzia, C. Meyer, T. Tuschl, The E3 ubiquitin ligase RNF10 modifies 40S ribosomal subunits of ribosomes compromised in translation. *Cell Rep.* **36**, 109468 (2021).

41. M. Narita, T. Denk, Y. Matsuo, T. Sugiyama, C. Kikuguchi, S. Ito, N. Sato, T. Suzuki, S. Hashimoto, I. Machová, P. Tesina, R. Beckmann, T. Inada, A distinct mammalian disome collision interface harbors K63-linked polyubiquitination of uS10 to trigger hRQT-mediated subunit dissociation. *Nat. Commun.* **13**, 6411 (2022).

42. A. Miścicka, A. G. Bulakhov, K. Kuroha, A. Zinoviev, C. U. T. Hellen, T. V. Pestova, Ribosomal collision is not a prerequisite for ZNF598-mediated ribosome ubiquitination and disassembly of ribosomal complexes by ASCC. *Nucleic Acids Res.* **52**, 4627–4643 (2024).

43. A. C. De La Cruz, G. Tisdale, E. Nakayama, Z. Huang, N. K. Sinha, R. Green, B. Wu, Single-protein/RNA imaging reveals ZNF598 as a limiting factor in resolving collided ribosomes. *EMBO J.* **44**, 5215–5232 (2025).

44. A. González, A. Covarrubias-Pinto, R. M. Bhaskara, M. Glogger, S. K. Kuncha, A. Xavier, E. Seemann, M. Misra, M. E. Hoffmann, B. Bräuning, A. Balakrishnan, B. Qualmann, V. Dötsch, B. A. Schulman, M. M. Kessels, C. A. Hübner, M. Heilemann, G. Hummer, I. Dikić, Ubiquitination regulates ER-phagy and remodelling of endoplasmic reticulum. *Nature* **618**, 394–401 (2023).

45. E. Fiskin, T. Bionda, I. Dikic, C. Behrends, Global analysis of host and bacterial ubiquitinome in response to *Salmonella* typhimurium infection. *Mol. Cell* **62**, 967–981 (2016).

46. N. A. Kulak, G. Pichler, I. Paron, N. Nagaraj, M. Mann, Minimal, encapsulated proteomic-sample processing applied to copy-number estimation in eukaryotic cells. *Nat. Methods* **11**, 319–324 (2014).

47. M. Komatsu, S. Waguri, M. Koike, Y.-S. Sou, T. Ueno, T. Hara, N. Mizushima, J.-I. Iwata, J. Ezaki, S. Murata, J. Hamazaki, Y. Nishito, S.-I. Iemura, T. Natsume, T. Yanagawa, J. Uwayama, E. Warabi, H. Yoshida, T. Ishii, A. Kobayashi, M. Yamamoto, Z. Yue, Y. Uchiyama, E.

Kominami, K. Tanaka, Homeostatic levels of p62 control cytoplasmic inclusion body formation in autophagy-deficient mice. *Cell* **131**, 1149–1163 (2007).

48. D. W. Huang, B. T. Sherman, R. A. Lempicki, Bioinformatics enrichment tools: Paths toward the comprehensive functional analysis of large gene lists. *Nucleic Acids Res.* **37**, 1–13 (2009).

49. D. W. Huang, B. T. Sherman, R. A. Lempicki, Systematic and integrative analysis of large gene lists using DAVID bioinformatics resources. *Nat. Protoc.* **4**, 44–57 (2009).

50. R. De Cegli, D. Carrella, D. Siciliano, G. Gambardella, G. Napolitano, C. Di Malta, A. Ballabio, D. Di Bernardo, TFEBexplorer: An integrated tool to study genes regulated by the stress-responsive Transcription Factor EB. *Autophagy Rep.* **1**, 295–305 (2022).
